# Virtual Reality and Serious Videogame-Based Instruments for Assessing Spatial Navigation in Alzheimer’s Disease: A Systematic Review of Psychometric Properties

**DOI:** 10.1007/s11065-024-09633-7

**Published:** 2024-02-26

**Authors:** Juan Pablo Sánchez-Escudero, Ana María Galvis-Herrera, David Sánchez-Trujillo, Laura Cristina Torres-López, Cole J. Kennedy, Daniel Camilo Aguirre-Acevedo, Mauricio A. Garcia-Barrera, Natalia Trujillo

**Affiliations:** 1 National College of Public Health, University of Antioquia, Antioquia, Colombia; 2 Department of Psychology, University of Antioquia, Antioquia, Colombia; 3 Department of Psychology & Institute on Aging and Lifelong Health, University of Victoria, Victoria, BC Canada; 4 School of Medicine, University of Antioquia, Antioquia, Colombia; 5 Atlantic Fellowship in Equity in Brain Health, Global Brain Health Institute, University of California, San Francisco, CA USA; 6 Stempel College of Public Health and Social Work, Florida International University, Miami, FL USA

**Keywords:** Alzheimer disease, Psychometric properties, COSMIN, Virtual reality, Systematic review, Serious games, Digital neuropsychology

## Abstract

Over the past decade, research using virtual reality and serious game-based instruments for assessing spatial navigation and spatial memory in at-risk and AD populations has risen. We systematically reviewed the literature since 2012 to identify and evaluate the methodological quality and risk of bias in the analyses of the psychometric properties of VRSG-based instruments. The search was conducted primarily in July–December 2022 and updated in November 2023 in eight major databases. The quality of instrument development and study design were analyzed in all studies. Measurement properties were defined and analyzed according to COSMIN guidelines. A total of 1078 unique records were screened, and following selection criteria, thirty-seven studies were analyzed. From these studies, 30 instruments were identified. Construct and criterion validity were the most reported measurement properties, while structural validity and internal consistency evidence were the least reported. Nineteen studies were deemed very good in construct validity, whereas 11 studies reporting diagnostic accuracy were deemed very good in quality. Limitations regarding theoretical framework and research design requirements were found in most of the studies. VRSG-based instruments are valuable additions to the current diagnostic toolkit for AD. Further research is required to establish the psychometric performance and clinical utility of VRSG-based instruments, particularly the instrument development, content validity, and diagnostic accuracy for preclinical AD screening scenarios. This review provides a straightforward synthesis of the state of the art of VRSG-based instruments and suggests future directions for research.

**Supplementary Information:**

The online version contains supplementary material available at 10.1007/s11065-024-09633-7.

## Introduction

Alzheimer’s disease (AD) is a neurodegenerative disease and the most common cause of dementia syndrome worldwide (Alzheimer’s Association, 2023). Neuropathological hallmark AD signs include neurofibrillary tangles, senile plaques, and neuronal loss. These changes are primarily due to the accumulation of abnormal proteins such as beta-amyloid and phosphorylated tau (Alzheimer’s Association, 2023; Jack et al., 2018). The progression of AD pathology is paralleled by the progression of cognitive symptoms, leading to a gradual functional decline and culminating in a clinical-pathological entity known as dementia by AD or Alzheimer’s dementia (Alzheimer’s Association, 2023). Given that the older age is one of the most significant risk factors for AD (Alzheimer’s Association, 2023), the rising median age of the global population due to shifts in birth and death rates and the consistent rise in life expectancy is leading to an increase in AD prevalence (Alzheimer’s Association, 2023; Levy et al., 2016). In North America alone, it is estimated that by 2050, 12.7 million individuals aged 65 years and over will be affected by the disease (Alzheimer’s Association, 2023). As the prevalence and incidence of AD continue to rise, the development of valid and reliable instruments for cognitive assessment and follow-up from the preclinical stage of the disease is crucial (Levy et al., 2016).

According to the updated National Institute on Aging and Alzheimer’s Association guidelines, AD is recognized as a continuum consisting of stages that reflect the progression of neurodegeneration and cognitive symptoms (Jack et al., 2018). While the presence of AD can be only determined by the presence of biomarkers (i.e., Aβ protein and tau protein), cognitive symptoms can be used to determine the staging of the disease (Jack et al., 2018). Thus, traditionally AD has been divided into three different stages: (1) the preclinical stage, also named cognitively unimpaired, where the neuropathological process has begun but no behavioral and cognitive symptoms can be detected; (2) the prodromal or early-stage symptomatic AD, often called mild cognitive impairment (MCI), where some cognitive changes can be detected with no interference in the daily level of independence; and the (3) dementia stage, where neurocognitive symptoms interfere with daily living activities (Jack et al., 2018; Sperling et al., 2011).

Despite the advantages in the detection of the preclinical stage of AD using biomarkers, the secondary effects and costs associated with the techniques used for their collection (e.g., lumbar puncture, positron emission tomography scans, functional magnetic resonance imaging) have promoted the search and identification of low cost, reduced secondary effect markers with acceptable diagnostic accuracy that help in the screening process of the at-risk population (Levy et al., 2016; Lizio, 2020; Park, 2022; Sabbagh & Blennow, 2019).

In recent years, the development of cognitive markers based on information and communication technologies has been proposed as a suitable solution to this need (Ben-sadoun et al., 2018; Boot, 2015; Sacco et al., 2019; Sperling et al., 2014; Tong et al., 2016). Specifically in the context of preclinical detection of AD, spatial navigation and spatial memory task assessment using digital neuropsychology technologies, such as virtual reality and serious games, have been explored as alternatives for the detection of at-risk populations (Bayahya et al., 2021; Coughlan et al., 2018; Park, 2022; Poos et al., 2021; Puthusseryppady et al., 2022).

### Spatial Navigation and Spatial Memory in AD

Spatial navigation is the ability to determine and maintain a route from the starting point to the goal using different strategies and sources of information (Coughlan et al., 2018; Gazova et al., 2012). In complement, spatial memory is a cognitive function that allows an individual to recall the location of objects in the space and their spatial relations (Bird & Burgess, 2009; Jacobs, 2003; Kolarik & Ekstrom, 2015). Together, spatial navigation and memory are crucial for building and using effectively a unified mental representation of environment known as cognitive maps (Burgess, 2006; Epstein et al., 2017). Both in humans and other mobile species, the encoding and organization of spatial information is described using two frames of reference: allocentric and egocentric (Iachini et al., 2009). The egocentric representation of the environment involves using self-position and self-motion cues relative to the environment to codify the individuals’ position and to set directions and distance to the goal (Coughlan et al., 2018; Iachini et al., 2009). In contrast, allocentric representations are independent of the self-position and do not change as the individual moves through space, relying on the landmarks in relation to each other instead to navigate (Iachini et al., 2009; Lester et al., 2017).

Neural correlates underlying spatial navigation and memory encompass several brain areas, forming a complex network for processing spatial information (Coughlan et al., 2018; Iachini et al., 2009; Jacobs, 2003). Structures in the medial temporal lobe, such as the hippocampus, entorhinal cortex, and parahippocampal cortex, support the cognitive map and are crucial for spatial navigation using allocentric representations (Colombo et al., 2017; Coughlan et al., 2018; Eichenbaum & Cohen, 2014). In contrast, egocentric spatial representations are primarily linked to the parietal lobe, particularly the medial and posterior parietal cortex, posterior cingulate, precuneus, and retrosplenial cortex (Coughlan et al., 2018). While traditional views have emphasized allocentric navigation as hippocampal-dependent, contemporary models highlight the role of the hippocampus and parahippocampal cortex in egocentric navigation and acknowledge the contributions of other structures in cognitive map formation (Eichenbaum & Cohen, 2014; Goodroe et al., 2018; Kunz et al., 2021). Effective navigation in daily life often requires the integration of multiple sources of information, including both allocentric and egocentric representations requiring engagement from the frontal lobes, caudate nucleus, and thalamus in addition to medial temporal lobe structures (Coughlan et al., 2018; Morganti et al., 2013).

Recent research has linked deficits in spatial navigation and spatial memory tasks to the widespread neurodegeneration in medial temporal, parietal, and frontal brain regions during AD progression (Coughlan et al., 2018; Jacobs, 2003; Laczó et al., 2018; Nedelska et al., 2012). Due to AD pathophysiology effects on areas such as the entorhinal cortex, hippocampus, posterior cingulate cortex, and precuneus (Coughlan et al., 2018, 2019), individuals with AD and MCI due to AD present deficits in tasks involving allocentric and egocentric navigation strategies (Coughlan et al., 2019; Laczó et al., 2014, 2018; Vlcek & Laczó, 2014) and spatial memory (Kessels et al., 2015; Mitolo et al., 2013). Notably, individuals with AD dementia and MCI due to AD exhibit impairments in egocentric strategies, a pattern not observed in the preclinical stages of AD where allocentric performance is severely impaired (Laczó et al., 2018; Nedelska et al., 2012). This difference in the progression of allocentric and egocentric representations has relevant conceptual and practical implications for developing valid and reliable instruments and tasks to detect AD at-risk populations as it states the basis for hypothesis specification and construct validation of instruments (Ritchie et al., 2018; Ruggiero et al., 2018).

### Tasks for Assessing Spatial Navigation in Humans

Since the concept of the cognitive map was first proposed by Tolman (1948) based on his work with rodents, researchers have adapted and designed different tasks to assess spatial navigation in humans in highly controlled settings such as laboratories (Burgess et al., 2004; Fernandez-Baizan et al., 2020a, 2020b; Schöberl et al., 2020). Paradigms such as the hidden goal task, a human version of the Morris water mazes for mice, have been successfully used for the assessment of allocentric and egocentric spatial navigation strategies, showing correlation with the brain structures implied in the spatial processing (Laczó et al., 2012; Nedelska et al., 2012).

Traditionally, tasks for assessing the egocentric and allocentric spatial memory adapt environments such as rooms or corridors to test specific hypotheses regarding spatial navigation strategies (Burgess et al., 2004; Fernandez-Baizan et al., 2019; Motes et al., 2006; Ribordy et al., 2013). While these environments provide high control of the experimental situation, they can have limited ecologic validity and are expensive and challenging to reproduce in research and clinical settings (Campbell et al., 2009; Vasser et al., 2017). To address these limitations, researchers over the last two decades have increasingly turned to digital technologies, such as virtual reality (Castegnaro et al., 2022; Diersch & Wolbers, 2019; Nguyen et al., 2023; Puthusseryppady et al., 2022; Ventura et al., 2013; Wiener et al., 2020) and serious games (Coughlan et al., 2019, 2020; Puthusseryppady et al., 2022), to assess human spatial navigation skills.

Virtual reality consists in the use of interactive computer-generated graphics for simulating realistic environments, creating a sensory experience with different immersion degrees where the user perceives the world through screen or head-mounted devices (Pan et al., 2006; Zheng et al., 1998). On the other hand, serious games are developed with purposes other than entertainment (Deterding et al., 2011). Recent advancements in digital technologies, access to virtual reality devices, 3D modeling software, and game engines have led researchers to design and adapt their own navigational tasks and protocols (Alsbury-Nealy et al., 2021; Laczó et al., 2021; Vasser et al., 2017; Wiener et al., 2020). Among the different adaptations, virtual scenarios can incorporate game elements, such as badges, leaderboards, avatars, and rewarding systems, adaptations which have resulted in the creation of virtual reality-based serious games (Manera et al., 2017; Tong et al., 2014, 2016).

Virtual reality is a flexible technology that enables the creation of diverse environments without or with few constraints (e.g., device memory and processor capacity can limit the number and quality of objects to render in a scene). In the context of spatial navigation and memory assessment, guided by non-human research, different types of mazes have been adapted for assessing allocentric and egocentric spatial navigation strategies (Lee et al., 2014; Nguyen et al., 2023). More recently, tasks involving route learning, path integration, wayfinding, and landmark placement have been adapted to virtual reality scenarios (Puthusseryppady et al., 2022; Wiener et al., 2020).

Although virtual reality and serious game-based (VRSG-based) instruments are developed following recommendations for software and experimental psychological task programming, aspects related to the quality of design and analysis of psychometric properties are not usually assessed using systematic criteria (Silva et al., 2023). Since test reliability and validity of the scores are prerequisites for robust assessment, analyzing the methodological quality of the studies on measurement properties is crucial for further advances in the field (Mokkink et al., 2018). In recent years, some other reviews have focused on the contrast between real-world and virtual reality tasks (Cogné et al., 2017; Tuena et al., 2021) or summarize the diagnostic accuracy for detecting mild cognitive impairment (MCI) and dementia (Chan et al., 2021; Molina da Costa et al., 2020). However, the systematic assessment of the methodological quality of studies on psychometric properties remains unattended so far.

Therefore, the specific objectives of this review were (1) identify and describe the existing VRSG-based instruments developed for assessing spatial navigation and spatial memory in samples with preclinical AD, MCI due to AD, and dementia by AD; (2) evaluate the methodological quality of the studies; and (3) establish the risk of bias in the analysis of psychometric properties of the instruments used in the studies identified using COSMIN guidelines. This review will provide information regarding the quality of existing evidence, offering researchers and clinical neuropsychologists a comprehensive analysis of state of the art regarding VRSG-based instruments for the neuropsychological assessment of spatial navigation and spatial memory in at-risk populations for AD. Based on the analyzed evidence, recommendations regarding using these instruments in clinical and research settings and future directions for advances in digital neuropsychology research are provided.

## Methods

A protocol was developed and registered with PROSPERO (CRD42022339039) in accordance with COSMIN guidelines, which established a procedure for the quality appraisal of instruments (Mokkink et al., 2018). Although the COSMIN methodology is originally focused on Patient-Reported Outcome Measures usage, it provides valuable and standardized criteria for evaluating other types of measurement instruments (Mokkink et al., 2018). Key components of COSMIN include a detailed checklist for assessing the methodological quality of studies for each measurement property and an emphasis on evaluating the generalizability and relevance of the study findings (Mokkink et al., 2018).

### Data Sources and Searches

A systematic literature search, initially conducted from July to November 2022 and subsequently updated in November 2023, aimed to identify published articles examining the psychometric properties of VRSG-based instruments for assessing spatial memory and navigation in AD. Eight databases were selected for sourcing peer-reviewed literature: Scopus, PubMed/Medline, EBSCO, APA PsycINFO, Web of Science, EMBASE, SciELO, and RedALyC. A supplementary gray literature review was implemented to encompass a broader range of relevant instruments. The extended search included ProQuest and LILACS for theses and dissertations, as well as PsycArticles, Google Scholar, WHO IRIS, Index Psicología–Periódicos, SciELO Preprints, and Open Science Framework for other gray literature sources. The results from these sources were subjected to the same rigorous analysis and eligibility criteria as those identified from peer-reviewed literature databases. These efforts aimed to capture literature in the early stages or in preparation for peer review and to reduce the influence of publication bias on our results (Winters & Weir, 2017). The search strategy also involved Spanish-language terms, which were identified through an initial search in the library systems and repositories of universities offering graduate programs in neuropsychology and related disciplines. See supplementary material for a detailed description of search equations.

### Study Selection

Studies met inclusion criteria if they (1) report original data on the analysis of psychometric properties of VRSG-based instruments for the assessment of spatial navigation or spatial memory; (2) involve participants with an increased risk for AD (i.e., genetic risk for sporadic AD, suspected MCI due to AD) or participants in the continuum of AD; (3) use gamification, serious games, or virtual reality to develop or adapt the instruments; (4) have accessibility of the full text; (5) are published in English or Spanish language, regardless of the original language used for developing the instrument; and (6) are published between 2012 and 2023. The date range was chosen to reflect the growing utilization of virtual reality-based assessments and technological advancements over the past decade and the recent developments in virtual reality technology and 3D modeling software on the regard of neuropsychological assessment (Cipresso et al., 2018; Diersch & Wolbers, 2019; Krohn et al., 2020).

Studies identified were entered into the Rayyan online reviewing system (Ouzzani et al., 2016) for screening. Then, the duplicated studies were removed. Four authors (J.P., A.G., D.S., and L.T.) screened titles and abstracts to determine whether the studies met the inclusion criteria. Each study was randomly assigned to a pair of independent reviewers for inclusion criteria assessment. Uncertainty about eligibility or data accuracy was resolved by consensus between reviewers. The criteria for exclusion described in Table 1 were used.

**Table 1:** Criteria for the study exclusion

**Criteria**	**Description**
Language	Paper published in a language other than Spanish or English
Outcome	The primary outcome studied is not AD or MCI
Population	The participants are not diagnosed with AD or MCI
Publication type	The paper is not an empirical research paper
Study design	Randomized clinical trials, systematic and literature review were excluded unless they specifically addressed the assessment of psychometric properties
No VRSG-based instrument	The study used a software-based measure, but there is no use of gamification strategies or virtual reality scenarios
Instrument modality	The study did not use a software-based instrument (e.g., paper–pencil test and real environment assessments were used)
Construct	The study does not present evidence on spatial navigation or spatial memory; instead, it analyzes other cognitive processes
Previously reported findings	The findings of this study have been documented in another peer-reviewed publication

### Data Extraction

Two independent reviewers extracted the relevant data for each study. Data extraction included study population characteristics (i.e., number of participants, age, gender, risk status for AD), publication details (i.e., authors, year of publication), instrument description, virtual reality or serious game technology, study design, neuropsychological correlates, and main findings on the psychometric properties (i.e., indicators for reliability such as internal consistency, test–retest, and indicators for content validity, criterion validity, and construct validity) extracted and exported in Microsoft Excel files following COSMIN guidelines (Prinsen et al., 2018).

Instrument development design and methodological quality of the included studies were evaluated separately in accordance with COSMIN guidelines (Prinsen et al., 2018). Psychometric properties (i.e., content validity, structural validity, internal consistency, cross-cultural validity/measurement invariance, reliability, measurement error, criterion validity, and construct validity) were analyzed when the original data was available. All psychometric properties were defined according to COSMIN guidelines. See supplementary material for a detailed description of the definition of measurement properties.

This review used a comprehensive approach to assess the analysis of psychometric properties in the studies identified. Thus, the performance differences between groups with different levels of risk for AD were considered evidence of construct validity (i.e., known-group validity), while correlations between instrument scores and neuropsychological and neurological measures were seen as evidence of convergent validity. Diagnostic accuracy measures were evaluated according to COSMIN guidelines for criterion validity. This approach allowed the review to obtain relevant information about the measuring properties, even if the authors did not explicitly present this information.

### Assessment of Psychometric Properties

The methodological quality of studies on measurement properties of VRSG-based instruments was assessed by following the risk of bias checklist and the quality judgment criteria contained in the ten-item COSMIN checklist (Mokkink et al., 2018). For each article, two independent reviewers assessed the methodological quality of the studies and the risk of bias within the psychometric properties reported by the authors. The results were rated as “very good,” “adequate,” “doubtful,” or “inadequate” according to the four-point rating system and the *worst score counts principle*, taking the lowest rating of any standard in the analysis of the psychometric property analyzed (Mokkink et al., 2018).

## Results

**Fig. 1:**
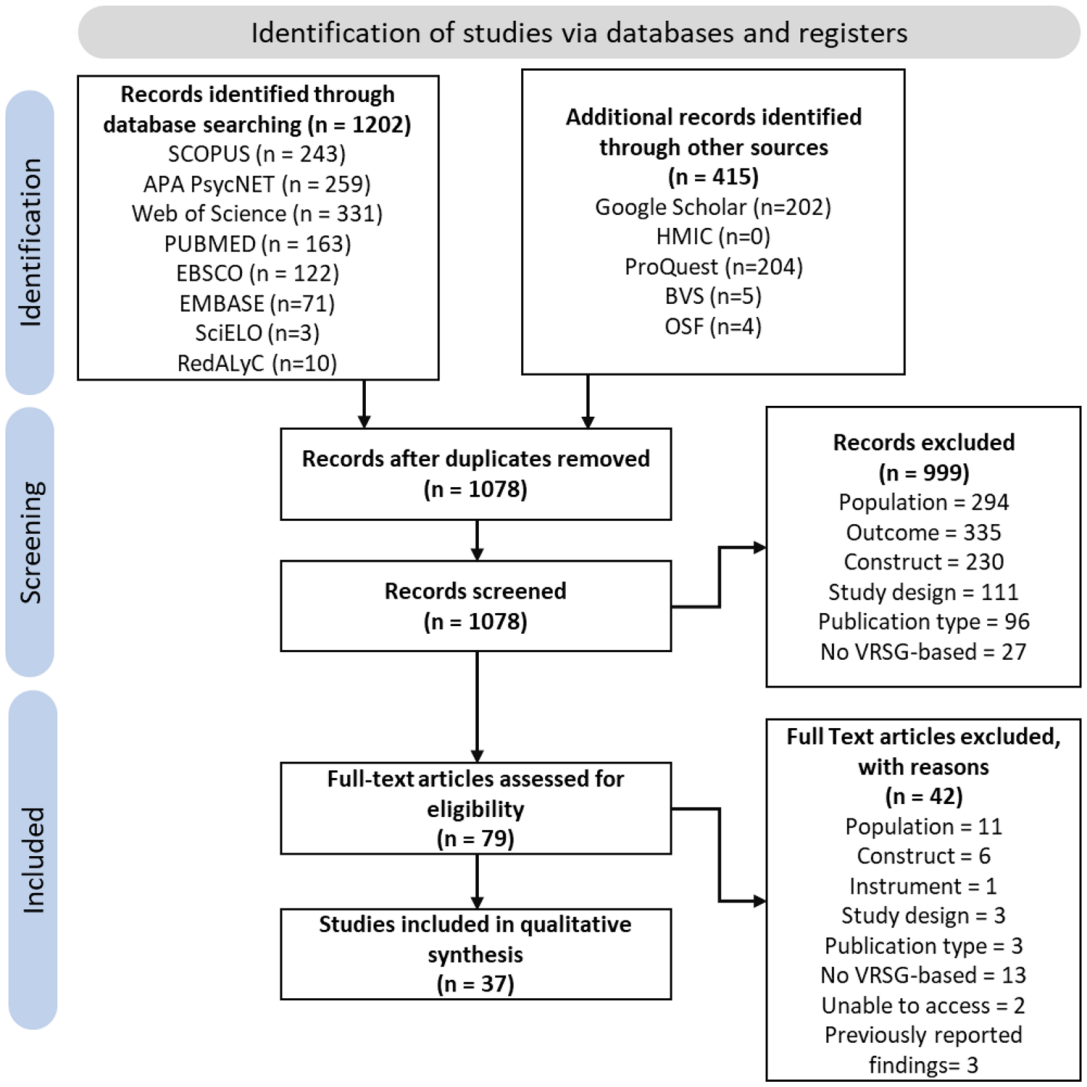
PRISMA flow diagram. Summary of search

The search strategy returned 1078 unique records, from which 79 were selected for full-text screening. Of these, 37 articles reported evidence about the psychometric properties of 30 instruments. As three of the included studies presented evidence on more than one instrument (Coughlan et al., 2020; Morganti et al., 2013; Puthusseryppady et al., 2022), COSMIN criteria were assessed in each VRSG instrument. Figure 1 shows the results of the search in the databases. Due to the eligibility criteria, pilot studies using healthy populations for developing the instrument were excluded. All studies identified described virtual reality-based instruments. Among these studies, ten of them included gamification strategies (Bayahya et al., 2021; Bierbrauer et al., 2020; Colmant et al., 2023; Coughlan et al., 2019, 2020; Gellersen et al., 2021; Lee et al., 2014; Pink et al., 2023; Puthusseryppady et al., 2022; Tarnanas et al., 2015).

### Study Summary and Assessment Methodology

A summary of the findings is presented in Table 2. Among the VRSG-based instruments identified, 13 (43.3%) were developed in English, while four instruments (13.3%) were developed in Italian. Additionally, two instruments (6.7%) each were developed in Czech, French, Greek, and Korean. At least one instrument (3.3%) was developed in each of Farsi, German, Portuguese, and Spanish, demonstrating the global diversity of these tools. Regarding the technology used, four instruments (13.3%) were applied on iPad devices (Coughlan et al., 2019, 2020; Puthusseryppady et al., 2022; Ritchie et al., 2018), whereas one was developed to be used on mobile phones (Tarnanas et al., 2015), and remaining instruments were developed to be applied using personal computers and VR devices. A description of the instruments is provided as supplementary material. Among the studies using VR, five used immersive modalities (Castegnaro et al., 2022; Da Costa et al., 2022; Moussavi et al., 2022; Tarnanas et al., 2015; Zen et al., 2013), where the examinee wears a head-mounted display to see while a sensor collects data about their movement and position (Morganti et al., 2013).

**Table 2:** Summary of studies

**Reference**	**VRSG-based instrument**	**Participants**					**Technology**	**Mode of administration**	**Language**	**Country**
		**Subgroups**	***n***	**Age**		**Sex**				
				**Mean**	**SD**	**(%female)**				
Bellassen et al. (2012)	The starmaze task	HC	20	28.2	5.7	55%	Virtual reality	Personal computer	French	France
		HC	19	48.3	6.8	47%				
		HC	24	72	4.2	54%				
		aMCI due to AD	14	70.8	8.8	29%				
		AD dementia	16	70	9.0	50%				
		FTD-FV	11	68.2	7.8	46%				
Caffò et al. (2012)	Virtual reorientation test (VReoT)	aMCI-SD	28	69.9	5.2	60%	Virtual reality	Personal computer	Italian	Italy
		aMCI-MD	23	73.9	4.7	65%				
		HC	53	68.1	6.0	55%				
Tarnanas et al. (2012)	Virtual museum system (VAP-M)	aMCI	25	78	4.7	72%	Virtual reality	Personal computer	Greek	Greece
		HC	25	77	4.9	80%				
Morganti et al. (2013)	VR-maze spatial task (VRMST)	HC	26	77.2	5.3	50%	Virtual reality	Personal computer	Italian	Italy
		AD dementia	26	81.0	6.3	50%				
	VR-road map task (VRMT)	HC	26	77.2	5.3	50%	Virtual reality	Personal computer	Italian	Italy
		AD dementia	26	81.0	6.3	50%				
Zen et al. (2013)	Virtual reality navigational experiment (VRN)	Probable AD dementia	8	71.1	11.8	50%	Virtual reality	Personal computer	English	Canada
		HC	11	70.4	4.3	45%				
Lee et al. (2014)	VRAM task	HC	20	70.8	5.2	55%	Virtual reality	Personal computer	Korean	South Korea
		Probable AD	20	72.4	5.6	50%				
		aMCI	20	70.7	5.0	45%				
Lesk et al. (2014)	Virtual reality for early detection of AD (VREAD)	HC	22	69.8	4.6	77%	Virtual reality	Personal computer	English	England
		MCI	9	72.7	6.6	44%				
	VRAM task	HC	20	70.8	5.2	55%	Serious game	Personal computer	Korean	South Korea
		Probable AD dementia	20	72.4	5.6	50%				
		aMCI	20	70.7	5.0	45%				
Kunz et al. (2015)	Object location memory task	ε3ε4 carriers	38	22.8	0.5	51%	Virtual reality	N/R	English	USA
		ε3ε4 carriers	37	22.3	0.5	53%				
Serino et al. (2015)	Virtual room environment	Probable AD dementia	15	83	5.6	27%	Virtual reality	Personal computer	Italian	Italy
		aMCI	15	78	5.5	27%				
		HC	15	74	7.4	40%				
Tarnanas et al. (2015)	Complex activities of daily living	HC	76	70.1	13.3	65%	Serious game	Mobile phone	Greek	Greece
		aMCI	85	72.6	10.1	62%				
		Early AD dementia	86	76.6	10.6	63%				
Allison et al. (2016)	Cognitive mapping task	HC biomarker +	12	73.0	9	58%	Virtual reality	Personal computer	English	USA
		HC biomarker −	42	69.0	9	55%				
		Early AD dementia	15	77.0	10	40%				
Migo et al. (2016)	Platform task	HC	10	70.3	6.5	40%	Virtual reality	Personal computer	English	England
		aMCI	8	69.6	5.8	38%				
Caffò et al. (2018)	Virtual reorientation test (VReoT)	HC	203	68.6	7.1	47%	Virtual reality	Personal computer	Italian	Italy
		aMCI SD	14	70.9	8	71%				
		aMCI MD	22	73	4.7	50%				
		aMCI MD	16	74.9	8	56%				
		naMCI MD	10	75.3	6.7	90%				
		Probable AD dementia	21	74.3	6.6	67%				
Konishi et al. (2018)	Concurrent spatial discrimination task (CSDLT)	ε3ε4 carriers	15	65.6	4.5	N/R	Virtual reality	Personal computer	English	Canada
		HC	49	66.5	4.6	N/R				
Mohammadi et al. (2018)	Virtual neighborhood task (VRNT)	HC	30	69.8	1.4	50%	Virtual reality	Personal computer	Farsi	Iran
		AD dementia	20	73.6	2.5	65%				
		aMCI SD	30	70.0	1.6	63%				
		aMCI MD	30	70.0	1.7	60%				
Parizkova et al. (2018)	Y-maze strategy assessment (yVSA)	HC	20	67.5	7.1	10%	Virtual reality	Personal computer	Czech	Czech Republic
		aMCI due to AD	28	74.5	5.9	64%				
		AD dementia	21	73.2	6.9	57%				
Ritchie et al. (2018)	Reality supermarket trolley task	Non-AD FH	81	54.2	10.8	70%	Virtual reality	Tablet	French	France
		AD FH	94	55.3	6.6	71%				
Serino et al. (2018)	Virtual room environment	Probable AD dementia	15	86	4.0	73%	Virtual reality	Personal computer	Italian	Italy
		HC	15	83	5.6	80%				
Allison et al. (2019)	Modified cognitive mapping task	ADRC	30	73	4	43%	Virtual reality	Personal computer	English	USA
		RPR	60	67	5	68%				
Coughlan et al. (2019)	Sea Hero Quest (SHQ)	e3e3 carriers	29	62.5	5.3	48%	Virtual reality	Tablet	English	England
		e3e4 carriers	31	62.3	6.2	64%				
		Benchmark players	27,108	58.9	5.9	51%				
Bierbrauer et al. (2020)	The apple game	ε3ε3 carriers	76	42.6	2.4	47.30%	Virtual reality	Personal computer	English	Belgium
		ε3ε4 carriers	23	46.1	4.2	52.10%				
Coughlan et al. (2020)	Virtual supermarket test (VST)	ε3ε3 carriers	33	64.4	5.6	44%	Virtual reality	Tablet	English	England
		ε3ε4 carriers	31	63.7	6.4	70%				
	Sea Hero Quest (SHQ)	ε3ε3 carriers	33	64.4	5.6	44%	Virtual reality	Tablet	English	England
		ε3ε4 carriers	31	63.7	6.4	70%				
Davis and Sikorskii (2020)	VR simulation of a large senior residence	Probable AD dementia	7	76.6	5.0	47%	Virtual reality	Personal computer	English	USA
		HC	8	75	1.2	50%				
Levine et al. (2020)	Cognitive mapping task	No progression	78	70.6	7.9	50%	Virtual reality	Personal computer	English	USA
		Progression	17	78.9	7.5	41%				
Bayahya et al. (2021)	The virtual scenario	Control	65	60	7.9	85%	Virtual reality	Personal computer	N/R	Saudi Arabia
		MCI	20	68.5	9.3	60%				
		Dementia	30	80	8.1	33%				
Gellersen et al. (2021)	Sea Hero Quest (SHQ)	ε3ε3 carriers	26	63.4	6.1	50%	Virtual reality	Tablet	English	England
		ε3ε4 carriers	20	64.8	6.8	25%				
		ε3ε4 carriers	3	63.3	0.6	33%				
Laczó et al. (2021)	Navigation test suite	HC	78	68.2	6.8	72%	Virtual reality	Personal computer	Czech	Czech Republic
		aMCI	75	71.5	7.4	75%				
		Mild AD dementia	66	75.9	8.1	66%				
Castegnaro et al. (2022)	The object location task	Young controls	53	24.1	3	59%	Virtual reality	Personal computer	English	England
		HC	24	68.8	5.7	67%				
		aMCI	23	71.7	7.5	57%				
Da Costa et al. (2022)	SOIVET (maze task and route task)	aMCI	19	73	6.5	68%	Virtual reality	Personal computer	Portuguese	Brazil
		HC	29	70	5.3	69%				
Laczó et al. (2022)	Navigation test suite	HC	30	68.7	5.8	70%	Virtual reality	Personal computer	Czech	Czech Republic
		Non-AD MCI	31	70.3	7.7	48%				
		aMCI due to AD	33	72.3	6.4	61%				
		Mild AD dementia	28	74.3	5.9	64%				
Moussavi et al. (2022)	Virtual reality navigational experiment (VRN)	Probable AD dementia	65	72.4	8.7	43%	Virtual reality	Personal computer	English	Canada
		MCI	20	72.9	7.9	35%				
Park (2022)	Spatial cognitive task based on virtual reality (SCT-VR)	aMCI	36	75.3	6.2	61%	Virtual reality	Personal computer	Korean	Korea
		HC	56	73.0	6.5	55%				
Puthusseryppady et al. (2022)	Virtual supermarket test (VST)	HC	21	68.4	7.6	56%	Virtual reality	Tablet	English	England
		Probable AD dementia	16	70.3	6.6	50%				
	Sea Hero Quest (SHQ)	HC	21	68.4	7.6	56%	Virtual reality	Tablet	English	England
		Probable AD dementia	16	70.3	6.6	50%				
Silva et al. (2023)	SOIVET (maze task and route task)	HC	24	71.3	5.6	63%	Virtual reality	Personal computer	Portuguese	Brazil
		MCI	19	72.3	5.6	63%				
Colmant et al. (2023)	The apple game	ε4 carriers	73	58.6	15.2	62%	Serious game	Personal computer	English	Belgium
		ε4 noncarriers	198	57.3	16.3	65%				
Pink et al. (2023)	Virtual reality-based path integration task	ε3ε4 carriers	31	43.1	17.1	64%	Virtual reality	Personal computer	German	Germany
		Controls	73	45.3	15.9	76%				
Plaza-Rosales et al. (2023)	Virtual Morris water navigation (VMWN)	MCI due to AD	9	76.7	6.2	78%	Virtual reality	Personal computer	Spanish	Chile
		HC	9	71.2	8.5	56%				

*HC* healthy control, *AD* Alzheimer’s disease, *MCI* mild cognitive impairment, *aMCI* amnestic mild cognitive impairment, *aMCI-SD* amnestic mild cognitive impairment single domain, *aMCI-MD* amnestic mild cognitive impairment multiple domain, *FTD-FV* frontotemporal dementia frontal variant, *naMCI* non-amnestic mild cognitive impairment, *N/R* not reported, *FH* familial history, *ADRC* sample from the Alzheimer Disease Research Center, *RPR* sample from the Washington University in St. Louis Research Participant Registry

### Psychometric Property Assessment and Level of Evidence

#### Overall COSMIN Assessment of Instruments

The overall results of the COSMIN assessments are shown in Table 3. Among all the instruments identified, the psychometric properties reported were structural validity, internal consistency, criterion validity, and construct validity. None of the instruments reported evidence for all these psychometric properties, revealing a gap in robust psychometric evidence for the measurement properties of instruments used across the literature. Regarding the general quality of the studies, three were deemed adequate or very good (Allison et al., 2016; Caffò et al., 2018; Coughlan et al., 2019). Given the innovative nature of VRSG-based instruments, the lack of pilot studies did not automatically lead to an inadequate or doubtful rating. Studies classified under these categories typically featured insufficient descriptions of their target populations, the constructs they intended to measure, or the theoretical frameworks underpinning these constructs (Mokkink et al., 2019).

**Table 3:** COSMIN assessment of instruments

**Reference**	**VRSG-based instrument**	**General quality**	**Structural validity**	**Internal consistency**	**Test–retest reliability**	**Criterion validity**	**Convergent validity**	**Known-group validity**
Bellassen et al. (2012)	The starmaze task	Inadequate	Adequate	NA	NA	Doubtful	NA	Adequate
Caffò et al. (2012)	Virtual reorientation test	Doubtful	NA	NA	NA	Very good	NA	Adequate
Tarnanas et al. (2012)	Virtual museum system	Inadequate	NA	NA	NA	NA	NA	Very good
Morganti et al. (2013)	VR-maze spatial task	Doubtful	NA	NA	NA	NA	Very good	Very good
	VR-road map task	Doubtful	NA	NA	NA	NA	Adequate	Adequate
Zen et al. (2013)	Virtual reality navigational experiment	Doubtful	NA	NA	NA	NA	NA	Doubtful
Lesk et al. (2014)	Virtual reality for early detection of AD	Inadequate	NA	NA	NA	NA	Doubtful	Doubtful
Lee et al. (2014)	VRAM task	Inadequate	NA	NA	NA	NA	Adequate	NA
Kunz et al. (2015)	Object location memory task	Inadequate	NA	NA	NA	NA	Very good	Very good
Serino et al. (2015)	Virtual room environment	Doubtful	NA	NA	NA	NA	NA	Very good
Tarnanas et al. (2015)	Complex activities of daily living	Doubtful	NA	NA	Adequate	NA	NA	Very good
Allison et al. (2016)	Cognitive mapping task	Adequate	NA	Very good	NA	Very good	NA	Very good
Migo et al. (2016)	Platform task	Doubtful	NA	NA	NA	NA	NA	Adequate
Caffò et al. (2018)	Virtual reorientation test	Adequate	NA	Very good	NA	NA	NA	Adequate
Konishi et al. (2018)	Concurrent spatial discrimination task	Doubtful	NA	NA	NA	NA	NA	Adequate
Mohammadi et al. (2018)	Virtual neighborhood task	Doubtful	NA	NA	NA	NA	NA	Doubtful
Parizkova et al. (2018)	Y-maze strategy assessment	Doubtful	NA	NA	NA	NA	Very good	Very good
Ritchie et al. (2018)	Reality supermarket trolley task	Inadequate	NA	NA	NA	NA	Very good	Doubtful
Serino et al. (2018)	Virtual reality-based procedure	Doubtful	NA	NA	NA	NA	NA	Doubtful
Allison et al. (2019)	Modified cognitive mapping task	Doubtful	Adequate	Very good	Adequate	Very good	NA	NA
Coughlan et al. (2019)	Sea Hero Quest	Very good	NA	NA	NA	Very good	NA	Very good
Bierbrauer et al. (2020)	The apple game	Adequate	NA	NA	NA	NA	Very good	Very good
Coughlan et al. (2020)	Virtual supermarket test	Inadequate	NA	NA	Doubtful	NA	NA	NA
	Sea Hero Quest	Inadequate	NA	NA	Doubtful	NA	NA	NA
Davis and Sikorskii (2020)	VR simulation of a large senior residence	Inadequate	NA	NA	NA	NA	NA	Doubtful
Levine et al. (2020)	Cognitive mapping task	Inadequate	NA	NA	NA	Very good	NA	NA
	Route learning task	Inadequate	NA	NA	NA	Very good	NA	NA
Bayahya et al. (2021)	The virtual scenario	Inadequate	NA	NA	NA	NA	NA	Doubtful
Gellersen et al. (2021)	Sea Hero Quest	Inadequate	NA	NA	NA	NA	NA	Very good
Laczó et al. (2021)	Navigation test suite	Inadequate	NA	NA	NA	NA	NA	Very good
Castegnaro et al. (2022)	The object location task	Inadequate	NA	NA	NA	Very good	Very good	Very good
Da Costa et al. (2022)	Maze task and route task (SOIVET)	Doubtful	NA	NA	NA	Very good	Doubtful	Adequate
Laczó et al. (2022)	Navigation test suite	Doubtful	NA	NA	NA	Very good	Very good	Very good
Moussavi et al. (2022)	Virtual reality navigational experiment	Doubtful	NA	NA	NA	NA	NA	Doubtful
Park (2022)	Spatial cognitive task based on virtual reality (SCT-VR)	Inadequate	NA	NA	Adequate	Very good	NA	Adequate
Puthusseryppady et al. (2022)	Virtual supermarket test	Inadequate	NA	NA	NA	NA	Adequate	Very good
	Sea Hero Quest	Inadequate	NA	NA	NA	NA	Adequate	Very good
Silva et al. (2023)	Maze task and route task (SOIVET)	Adequate	NA	NA	Adequate	NA	NA	NA
Colmant et al. (2023)	The apple game	Adequate	NA	NA	NA	NA	NA	Very good
Pink et al. (2023)	Virtual reality-based path integration task	Adequate	NA	NA	NA	NA	NA	Doubtful
Plaza-Rosales et al. (2023)	Virtual Morris water navigation	Doubtful	NA	NA	NA	NA	NA	Very good

*NA* not assessed

#### Structural Validity

Two studies (5.4%) reported evidence of structural validity (Allison et al., 2019; Bellassen et al., 2012). One of the studies (Bellassen et al., 2012) used exploratory factor analysis rather than confirmatory factor analysis required to be classified as very good according to the COSMIN guidelines (Mokkink et al., 2018). However, for both studies, no sample size calculation or rationale was presented, leading to concerns about the sample size adequacy for the analyses.

#### Internal Consistency

Three studies (8.1%) reporting the internal consistency of two instruments were identified (Allison et al., 2016, 2019; Caffò et al., 2018). The internal consistency for the *virtual reorientation test* was 0.79 (Caffò et al., 2018); Cronbach’s alpha was calculated using the subtask score as the units of measurement. Additionally, the internal consistency of the *cognitive mapping task* was reported differently between the studies (Allison et al., 2016, 2019). Both studies reported on the internal consistency of the wayfinding task, which comprises a learning phase, where participants explored a virtual environment and placed an “X” at all landmark locations on a blank 2D map, and a retrieval phase, where participants completed a series of supplementary tasks (i.e., landmark free recall, landmark location memory, and landmark identification memory) (Allison et al., 2016, 2019).

In the first study (Allison et al., 2016), Cronbach’s alpha was calculated using scores from the supplementary tasks, resulting in a reported consistency of *α* = 0.86. For the modified version (Allison et al., 2019), the internal consistency was calculated separately. For continuous scores, Cronbach’s alpha was used, resulting in the learning phase (*α* = 0.83) being reported as consistent, in contrast to retrieval phase (*α* = 0.35) which presented a low consistency. Kuder Richardson-20 was used for dichotomic scores, resulting in different levels of consistency for the landmark identification (KR_20_ = 0.87), scene recognition (KR_20_ = 0.87), and free recall (KR_20_ = 0.62) subtasks (Allison et al., 2019).

#### Reliability (Test–Retest)

Four studies (10.8%) reporting evidence about the test–retest reliability of five instruments were identified (Allison et al., 2019; Coughlan et al., 2020; Park, 2022; Tarnanas et al., 2015). The quality of evidence was deemed as very good in one study (Tarnanas et al., 2015), adequate in another (Allison et al., 2019), and doubtful in the remaining two (Coughlan et al., 2020; Park, 2022). The recall period varied between 2 weeks (Park, 2022) and 18 months (Coughlan et al., 2020), with 3 months being the most frequent (Allison et al., 2019; Tarnanas et al., 2015). No rationale for the selection of the recall period was provided in any of the studies.

The test–retest reliability for the *spatial cognitive task* based on virtual reality (Park, 2022) was high (ICC = 0.982, *p* < 0.001). In the case of the *virtual supermarket* (Coughlan et al., 2020), the test–retest reliability was moderate to adequate, depending on the subtask. Thus, the reliability of the egocentric score was adequate (ICC = 0.72), whereas the heading subtask was moderate (ICC = 0.50). For the *Sea Hero Quest* (Coughlan et al., 2020), the test–retest reliability was moderate for both distance (ICC = 0.50) and duration (ICC = 0.48) scores.

The accuracy-based tasks for the *complex activities of daily living* (Tarnanas et al., 2015) showed fair to moderate reliability (ICC 0.33–0.57), whereas latency-based measures exhibited good to excellent reliability measures (ICC 0.69–0.85). The *modified cognitive mapping task* (Allison et al., 2019) showed adequate reliability. Specifically, the tasks learning phase (ICC = 0.719), free recall (ICC = 0.570), and landmark identification (ICC = 0.722) exhibited good reliability. Effects of recall period on performance are yet to be determined.

#### Criterion Validity

Ten studies (27%) reporting evidence about the criterion validity and diagnostic accuracy of 11 instruments were identified. Nine studies were deemed very good as they calculated the area under the ROC curve for the continuous scores. Seven studies determined the sensitivity and specificity of the instruments based on the cutoff score estimated by Youden’s index (Allison et al., 2016, 2019; Bellassen et al., 2012; Da Costa et al., 2022; Laczó et al., 2022; Levine et al., 2020; Park, 2022).

The studies showed a heterogeneous selection of the standard of reference. Clinical and neuropsychological assessments were used to determine the diagnostic status of the participants in nine studies (90%), while one study (Coughlan et al., 2019) used the APOE risk status determined by genotyping techniques as the standard of reference (see supplementary material for additional information regarding the diagnostic accuracy). Additionally, six studies employed biomarkers to complement the diagnosis (Allison et al., 2016, 2019; Castegnaro et al., 2022; Coughlan et al., 2019; Laczó et al., 2022; Levine et al., 2020).

#### Construct Validity (Convergent Validity)

Nine studies (24.3%) reported data about the correlation between software-based instruments and neuropsychological or neurological measures, constituting evidence of convergent validity according to COSMIN guidelines (Castegnaro et al., 2022; Da Costa et al., 2022; Kunz et al., 2015; Laczó et al., 2022; Lee et al., 2014; Lesk et al., 2014; Morganti et al., 2013; Parizkova et al., 2018; Ritchie et al., 2018). Of the studies reported, eight were deemed as very good (Bierbrauer et al., 2020; Castegnaro et al., 2022; Laczó et al., 2022; Morganti et al., 2013; Parizkova et al., 2018; Ritchie et al., 2018), two as adequate (Lee et al., 2014; Morganti et al., 2013), and the three remaining as doubtful (Da Costa et al., 2022; Lesk et al., 2014).

Neurological correlates were reported in four studies (Bierbrauer et al., 2020; Kunz et al., 2015; Laczó et al., 2022; Parizkova et al., 2018). The brain activity related to structures implicated in the visuospatial processing as the grid cells in the entorhinal cortex (Bierbrauer et al., 2020; Kunz et al., 2015), as well as the hippocampal volume (Laczó et al., 2022; Parizkova et al., 2018), showed negative correlations with the performance on tasks from the *object location memory task* (Kunz et al., 2015), the *apple game* (Bierbrauer et al., 2020), the *yVSA* (Parizkova et al., 2018), and the *navigation test suite* (Laczó et al., 2022).

Neuropsychological correlates were reported in five studies (Da Costa et al., 2022; Lee et al., 2014; Lesk et al., 2014; Morganti et al., 2013; Ritchie et al., 2018). The global score of cognitive functioning scales was reported in four studies (Da Costa et al., 2022; Lesk et al., 2014; Morganti et al., 2013; Ritchie et al., 2018). The *MMSE, Dementia Risk Score, and Addenbrooke’s Cognitive Examination* were used in three studies (Da Costa et al., 2022; Lesk et al., 2014; Morganti et al., 2013). The *Corsi Test* scores (i.e., forward, backward, and supraspan) (Morganti et al., 2013), the *Manikin test* (Morganti et al., 2013), the *Rey Complex Figure Test* (Lee et al., 2014), the *Money Road-Map* test (Da Costa et al., 2022), and *Benton’s Judgment of Line Orientation* test (Da Costa et al., 2022) were reported as visuospatial correlates. The *VR-maze spatial task* (Morganti et al., 2013), *VR-road map task* (Morganti et al., 2013), *VRAM task* (Lee et al., 2014), and the *SOIVET* task (Da Costa et al., 2022) showed a positive and statistically significant correlation with the visuospatial measures. Correlation coefficient values between VRSG-based instrument and neuropsychological tests are provided in supplementary material.

#### Construct Validity (Known-Group Validity)

Based on previous literature (Fernandez-Baizan et al., 2020a, 2020b; Laczó et al., 2021; Levine et al., 2020; Nedelska et al., 2012; Parizkova et al., 2018), a set of a priori hypotheses were established to analyze the construct validity through known-group comparison. Thus, according to the natural progression of AD and previous findings (Allison et al., 2016; Colombo et al., 2017; Coughlan et al., 2018), it was expected that older participants, and those in a more advanced stage of the disease, would perform worse than younger participants and those in preclinical or early symptomatic stages of AD. In addition, given the neurophysiological changes occurring in the preclinical stage of the disease, it was expected that participants with positive biomarkers for AD and a genetic risk factor for sporadic AD would exhibit worse performance than healthy controls (Allison et al., 2016; Coughlan et al., 2018).

Thirty-two studies (86.5%) reporting evidence on comparing subgroups of 29 instruments were identified. Seventeen analyses were deemed as very good in quality (Allison et al., 2016; Bierbrauer et al., 2020; Castegnaro et al., 2022; Colmant et al., 2023; Coughlan et al., 2019; Gellersen et al., 2021; Kunz et al., 2015; Laczó et al., 2021, 2022; Morganti et al., 2013; Parizkova et al., 2018; Plaza-Rosales et al., 2023; Puthusseryppady et al., 2022; Serino et al., 2015; Tarnanas et al., 2012, 2015), nine as adequate (Bellassen et al., 2012; Caffò et al., 2012, 2018; Da Costa et al., 2022; Konishi et al., 2018; Migo et al., 2016; Mohammadi et al., 2018; Morganti et al., 2013; Park, 2022), and eight as doubtful (Bayahya et al., 2021; Davis & Sikorskii, 2020; Lesk et al., 2014; Moussavi et al., 2022; Pink et al., 2023; Ritchie et al., 2018; Serino et al., 2018; Zen et al., 2013). The mean size of the groups compared was 34 subjects, while the mean age was 69.24 (± 12.7).

Evidence about the difference in the performance between high and low genetic risk for sporadic AD (i.e., APOE ε3ε3, APOE ε3ε4) was reported in six studies (Bierbrauer et al., 2020; Colmant et al., 2023; Coughlan et al., 2019, 2020; Gellersen et al., 2021; Kunz et al., 2015) and against healthy controls in two studies (Konishi et al., 2018; Pink et al., 2023). Differences in the navigational performance were reported in the four studies, even in the absence of differences in object location memory (Bierbrauer et al., 2020; Colmant et al., 2023; Kunz et al., 2015), memory binding (Gellersen et al., 2021), and path integration tasks (Castegnaro et al., 2022; Coughlan et al., 2019, 2020; Kunz et al., 2015). Spatial navigation alterations seem to be related to the wayfinding ability (Coughlan et al., 2019, 2020; Gellersen et al., 2021; Kunz et al., 2015). This difference in the navigational performance was also reported when the performance between APOE ε3ε4 carriers and healthy controls was compared in the *concurrent spatial discrimination task* (Konishi et al., 2018) and the *apple game* (Colmant et al., 2023).

The performance of healthy controls was compared with the AD and MCI population in 14 studies (Bellassen et al., 2012; Davis & Sikorskii, 2020; Laczó et al., 2021, 2022; Lee et al., 2014; Mohammadi et al., 2018; Morganti et al., 2013; Parizkova et al., 2018; Puthusseryppady et al., 2022; Serino et al., 2015, 2018; Silva et al., 2023; Tarnanas et al., 2015; Zen et al., 2013). As expected, healthy controls showed a better performance in tasks involving planning a path in the presence of allocentric and egocentric perspectives, such as the *VR-maze spatial* task and *virtual reality navigational* experiment (Morganti et al., 2013). Impairment in allocentric and egocentric spatial strategies has been reported in early-stage symptomatic AD and early dementia due to AD samples in multiple virtual reality-based instruments (Morganti et al., 2013; Parizkova et al., 2018; Serino et al., 2015; Zen et al., 2013). Specifically, allocentric impairment has been reported from MCI stages, while egocentric impairments from early AD (Morganti et al., 2013; Parizkova et al., 2018; Ritchie et al., 2018; Ruggiero et al., 2018).

## Discussion

Over recent years, technologies such as virtual reality and serious games have progressively expanded in the public health domain (Manera et al., 2017). Using simulated 3D environments to assess spatial navigation and spatial memory is a profitable methodology for accessing clinical samples, such as individuals within the AD continuum (Caffò et al., 2012). This review has identified and evaluated the methodological quality of 30 VRSG-based instruments using the COSMIN framework. Most studies have primarily examined criterion and convergent validity using cross-sectional designs, providing valuable evidence about the diagnostic accuracy and construct validity of the instruments. However, the reliance on such designs, coupled with the use of non-probabilistic sampling methods, potentially introduces selection and spectrum biases in the results (Knottnerus & Buntinx, 2009; Kohn et al., 2013; Pepe, 2003). This limitation, along with the heterogeneity of theoretical frameworks, complicates the interpretation of findings and hampers the broader clinical application of these instruments. These findings warrant a detailed discussion of the results and implications for future research.

Our findings are in agreement with previous systematic reviews, supporting that VRSG-based instruments demonstrate satisfactory diagnostic accuracy in distinguishing individuals with MCI and AD dementia from those with normal aging (Chan et al., 2021; Molina da Costa et al., 2020; Tuena et al., 2021). While the identified VRSG-based instruments demonstrate strengths in terms of construct measurement and diagnostic accuracy, some questions regarding their content validity and research design have raised broader questions about the theoretical framework and the standardization of development procedures. In the following sections, some of these aspects will be discussed in detail, aiming to identify strategies and future directions that can enhance the existing instruments and facilitate the development of new, more reliable, and valid VRSG-based instruments.

### Construct Validity of VRSG-Based Instruments

Construct validity refers to the degree to which the scores of an instrument are consistent with hypotheses, assuming that the instrument validly measures the intended construct (Mokkink et al., 2019). The evaluation of construct validity typically can be investigated using two key procedures: convergent validity and known-group validity (Mokkink et al., 2019). Convergent validity refers to determine the relations with other measures of good quality that are intended to assess the same construct (Mokkink et al., 2019). On the other hand, known-group validity refers to testing the instrument ability to distinguish between groups that are expected to differ based on the construct being measured (Mokkink et al., 2018). Both forms of validity require a precise and clear definition of the construct for formulating accurate hypotheses and selecting appropriate instruments for evaluation (Abma et al., 2016).

Multiple definitions supporting the development of VRSG-based instruments were identified. Constructs such as spatial navigation (Allison et al., 2016; Gellersen et al., 2021; M. Laczó et al., 2022; Levine et al., 2020; Migo et al., 2016; Puthusseryppady et al., 2022; Serino et al., 2015; Tarnanas et al., 2015), spatial memory (Coughlan et al., 2020; Konishi et al., 2018; Park, 2022), spatial cognition (Tarnanas et al., 2012), spatial reorientation (Caffò et al., 2012, 2018), topographic memory (Lesk et al., 2014), object location memory (Kunz et al., 2015), and allocentric and egocentric spatial strategies (Allison et al., 2019; Caffò et al., 2018; Mohammadi et al., 2018; Morganti et al., 2013) were reported as the conceptual basis supporting the VRSG-based instruments.

Despite the heterogeneity in definitions underlying VRSG-based instruments, most of them can be traced back to the cognitive map hypothesis (Epstein et al., 2017; O’Keefee & Nadel, 1978; Tolman, 1948), according to which the brain builds unified representations of the environment using spatial cues integrated within allocentric and egocentric frameworks (Epstein et al., 2017). Cognitive maps support navigational behavior, including path integration and wayfinding tasks, such as those included in *Sea Hero Quest* (Coughlan et al., 2019; Gellersen et al., 2021; Puthusseryppady et al., 2022), *object location task* (Castegnaro et al., 2022), *VR maze spatial task* (Morganti et al., 2013), *virtual neighborhood task* (Morganti et al., 2013), *virtual supermarket test* (Coughlan et al., 2020; Puthusseryppady et al., 2022), *cognitive mapping task* (Allison et al., 2016; Levine et al., 2020), *route learning task* (Levine et al., 2020), and the *navigation test suite* (Laczó et al., 2021, 2022).

The cognitive map hypothesis provides a comprehensive framework for comprehending the alterations in spatial navigation behavior across the AD continuum, as these changes are closely linked to the neurodegenerative progression of the condition (Coughlan et al., 2018). Extensive research in rodents and humans supports that specific groups of cells in the medial temporal lobe, such as the place, grid, boundary, and head direction cells, play a crucial role in forming these maps (Epstein et al., 2017).

This notion is highlighted in four studies (Bierbrauer et al., 2020; Kunz et al., 2015; Laczó et al., 2022; Parizkova et al., 2018) that show evidence regarding the relationship between the *apple game*, *object location memory task*, *navigation test suite*, *yVSA*, and neuronal activity. The structures included embrace grid cells and volumetry of areas such as the hippocampus and the entorhinal cortex, which are critical for forming cognitive maps (Coughlan et al., 2018; Epstein et al., 2017). This relationship supports that the cognitive map hypothesis is helpful in preclinical AD assessment, based on the evidence that structural changes in crucial areas in the hippocampal formation during the first stages of AD pathology could affect spatial navigation strategies. Specifically, allocentric impairment (Laczó et al., 2018; Nedelska et al., 2012) could be the first cognitive change caused by AD pathology, driven by tau-related degeneration in the medial temporal lobe rather than amyloid deposition in other brain areas such as the medial temporal lobe (Ritchie et al., 2018). Therefore, maintaining the distinction between allocentric and egocentric frameworks while also considering the neuronal progression of the disease could help to develop new cognitive tests that are highly sensitive to the presence of tauopathy in the medial temporal lobe, recognized as a distinctive characteristic of AD (Jack et al., 2018).

The relationship between VRSG-based instruments and neuropsychological tests encompasses various measures of visuospatial processing, such as the *Corsi Test* (Lee et al., 2014; Morganti et al., 2013), the *Complex Figure of Rey-Osterrieth* (Lee et al., 2014), the *Manikin test* (Morganti et al., 2013), the *Money Road-Map test* (Da Costa et al., 2022), and *Benton’s Judgment of Line Orientation test* (Da Costa et al., 2022). Additionally, measures of general cognitive status (Da Costa et al., 2022; Lesk et al., 2014; Morganti et al., 2013; Ritchie et al., 2018) have been reported to correlate with VRSG-based instruments. These correlations suggest that VRSG-based instruments are likely related to the spatial cognition domain, involving different spatial abilities such as egocentric mental rotation of space, spatial orientation and transformation, and spatial memory (Carter & Woldstad, 1985; Kessels et al., 2008, 2010; Vingerhoets et al., 1996). Furthermore, the performance on VRSG-based instruments may indicate cognitive detriment as AD progress. To enhance the interpretation of findings in terms of construct validity analyses, future research should report the purpose of correlation analysis.

Regarding the comparison between well-known groups, participants in the MCI stage demonstrated differences in performance compared to healthy controls, affirming the sensitivity of the intended construct in capturing early cognitive impairments. Additionally, including participants with genetic risk for AD further supported the discriminative capacity of the measures (Gellersen et al., 2021). Notably, healthy controls consistently outperformed impaired individuals and those at genetic risk, indicating the progressive nature of spatial navigation and spatial memory impairments along the AD continuum (Castegnaro et al., 2022; Da Costa et al., 2022; Laczó et al., 2021, 2022; Parizkova et al., 2018). However, differences in the classification criteria and metrics used among the studies impose some limitations in comparing results across the literature. The implications derived from the variation in criteria selection are discussed in the subsequent section.

### Criterion Validity of VRSG-Based Instruments

Criterion validity is the degree to which the score of an instrument adequately reflects a standard of reference (Mokkink et al., 2018). Therefore, the standard of reference selection should be carefully considered when interpreting its further use in the clinical setting (Liu et al., 2015; Pepe, 2003). The different standards of reference selected in the VRSG-based instruments that evaluated criterion validity limit the comparison of their results. For instance, while *Sea Hero Quest* (Coughlan et al., 2019) showed to be helpful in discriminating between subjects with and without a genetic risk for sporadic AD (i.e., APOE ε3ε4 carriers vs non-carriers), the *object location* task (Castegnaro et al., 2022), *SOIVET* (Da Costa et al., 2022), *navigation test suite* (Laczó et al., 2022), *SCT-VR* (Park, 2022), and the *VReoT* (Caffò et al., 2012) showed an adequate capacity to discriminate between MCI and healthy participants.

In the context of AD research, different sources of evidence are needed to determine the presence of the disease and its stage (Jack et al., 2018). According to the National Institute on Aging and Alzheimer’s Association research framework, both Αβ and pathologic tau biomarkers are required for the neuropathologic diagnosis of AD (Jack et al., 2018). In addition, cognitive symptoms are used only to stage the severity of the disease and should not be used to define the presence of AD (Jack et al., 2018).

Five studies presented evidence regarding the criterion validity using biomarker status as a reference (Allison et al., 2016, 2019; Castegnaro et al., 2022; Laczó et al., 2022; Levine et al., 2020). However, four collected Αβ and tau biomarkers (Allison et al., 2019; Castegnaro et al., 2022; Laczó et al., 2022; Levine et al., 2020) and these were used to define the standard of reference categories in two (Castegnaro et al., 2022; Laczó et al., 2021). In the remaining studies, criterion validity was tested using different categories derived from biomarkers such as high/low level of Αβ biomarker or clinical assessment (Allison et al., 2016, 2019; Levine et al., 2020). Therefore, only the *object location memory* task (Castegnaro et al., 2022) and the *navigation test suite* (Laczó et al., 2022) have shown validity to distinguish between MCI due to AD and healthy controls based on biomarkers. Further research is needed to analyze the criterion validity of VRSG-based instruments to discriminate between preclinical samples that are cognitively unimpaired according to currently available neuropsychological tests and those with positive biomarkers and healthy controls.

### Content Validity and Research Design Requirements

Content validity, which encompasses face validity, is the degree to which the instrument adequately accurately represents the intended construct it aims to measure, as determined by experts’ or potential users’ assessment (Terwee et al., 2018). Usually, the agreement among experts or pilot studies is accepted as evidence of this psychometric property (Mokkink et al., 2019).

In the absence of agreement among experts, ten of the identified studies reported pilot or previous studies (Allison et al., 2019; Caffò et al., 2012; Davis & Sikorskii, 2020; Laczó et al., 2022; Levine et al., 2020; Parizkova et al., 2018; Puthusseryppady et al., 2022; Ritchie et al., 2018; Tarnanas et al., 2012; Zen et al., 2013). Pilot studies provide valuable evidence for designing or refinishing characteristics of the instruments, such as the number of trials, learning or fatigue effects, and the total duration of the task, as shown by the pilot study of the *virtual reality navigational experiment* (Zen et al., 2013) and the *modified cognitive mapping task* (Allison et al., 2016).

While the COSMIN checklist lacks standardized criteria for assessing usability, it is pertinent to discuss this aspect due to the digital nature of the VRSG-based instruments. Usability, referring to the ease of use and effectiveness of a digital test or tool (Latendorf et al., 2021), is crucial for face validity, particularly in the context of digital interfaces. Using peripherals such as keyboards, joysticks, or gamepads adds an extra layer of difficulty in interacting with the instruments. This interaction should be as clear and easy as possible to prevent any affectation to the performance induced by an unfamiliar usage of the interface or peripheral, as suggested by the literature on recommendations for developing serious games and virtual reality tools for neurodegenerative diseases (Ben-sadoun et al., 2018; Manera et al., 2017). Most of the identified studies reported having included training or practice trials, with the exception of four studies which did not report this information (Davis & Sikorskii, 2020; Gellersen et al., 2021; Kunz et al., 2015; Zen et al., 2013).

In most of the studies reviewed, practice trials, following verbal or written instructions, were employed to familiarize participants with the requirements of the tasks and the use of peripherals. This strategy aimed to ensure a minimum proficiency level, mitigating performance disparities due to difficulties with device handling or task comprehension. While some studies provided usability metrics, such as the number and duration of practice trials, these metrics were not uniformly reported. Furthermore, the performance quality in practice sessions was used as a feasibility criterion, excluding participants who still needed to meet a baseline level of understanding or performance (Castegnaro et al., 2022; Morganti et al., 2013).

Although these measures provide insights into data quality and participant performance, there is a pressing need for further research to assess task and tool usability across the AD continuum. Such studies, particularly at the population level, remain uncommon. Notably, only Silva et al. (2023) presented evidence on the applicability of the *SOIVET* for participants with MCI due to AD, reporting no significant differences in cybersickness symptoms or immersion levels between MCI participants and healthy controls. To comprehensively measure usability, future studies should also consider user experience aspects like perceived usability (Lewis, 2018) and digital ergonomics (Ben-sadoun et al., 2018), incorporating methodologies tailored for user experience assessment (Sauro & Lewis, 2012).

In future studies, spectrum bias in the usability of the instrument should also be considered if pilot studies are carried out using cognitively unimpaired samples. This bias arises as the healthy population may differ in age, educational level, or technology literacy from the preclinical or at-risk population. Therefore, to mitigate potential spectrum bias in usability, especially when pilot studies use cognitively unimpaired samples, it is crucial to ensure that the target population perceives the instrument as usable. For this, we recommend considering the inclusion of usability questionnaires or focus group methodology and structured interviews in the pilot study (Ben-sadoun et al., 2018; Sauro & Lewis, 2012). The inclusion of such methodologies is recommended to comprehensively assess the usability of VRSG-based instruments, thereby enhancing their applicability in broader settings.

### Implications for Clinical Practice

The development of medical tests for screening and diagnosis typically follows a multiphase approach (Knottnerus & Buntinx, 2009; Pepe, 2003; Sackett & Haynes, 2002). The first phases in diagnostic research could be described as exploratory investigations typically following a case–control design, sometimes described as case-referent, and using non-probabilistic samples (Knottnerus & Buntinx, 2009; Pepe, 2003). Usually, these studies are described as phase one or phase two diagnostic studies and try to answer whether the test results in known affected patients differ from those in normal individuals (Sackett & Haynes, 2002). Despite their exploratory nature, this kind of diagnostic research studies provide valuable information, including sensitivity, specificity, likelihood ratios, discriminative capacity (Knottnerus & Buntinx, 2009; Sackett & Haynes, 2002), and usability (Ben-sadoun et al., 2018).

Our findings indicate that VRSG-based instruments align better with early stages of diagnostic research, specifically phases one and two (Sackett & Haynes, 2002). Consequently, their application in clinical screening of cognitive markers related to AD in their current state is not recommended. The limited evidence for diagnostic utility at this stage is primarily due to methodological constraints, such as non-representative sampling and variable reference standards (Pepe, 2003). To establish their efficacy in screening cognitive impairments associated with AD, further research must focus on whether performance in spatial navigation and memory tasks distinctly distinguishes between diverse groups with varying levels of risk for developing AD (Pepe, 2003). Additionally, generating normative data that accounts for variables such as age, education level, and sociodemographic variables is essential for providing an empirical context for interpreting individual performance on VRSG-based tasks (delCacho-Tena et al., 2023). The availability of normative data for VRSG-based instruments could support clinical integration and standardized use in population-level screening programs.

Longitudinal studies are imperative to track spatial navigation in at-risk or diagnosed individuals and should aim to identify dependable diagnostic markers and to evaluate the long-term health outcomes of VRSG-based assessments. Additionally, research into the cost-effectiveness of integrating these technologies into clinical settings is imperative to assess their feasibility and potential for widespread application. These directions establish a route for the development of fully usable VRSG-based instruments for the clinical screening of cognitive markers related to AD in clinical settings according to diagnostic research recommendations (Knottnerus & Buntinx, 2009; Pepe, 2003).

Despite these challenges, VRSG-based instruments represent the best opportunity for developing an integrated framework for neuropsychological assessment of spatial navigation and spatial memory in humans (Colombo et al., 2017; Coughlan et al., 2019; Park, 2022; Zucchella et al., 2014). Some identified VRSG-based instruments provide a platform for developing cognitive markers usable and translatable to clinical practice in the upcoming years. Instruments such as *Sea Hero Quest* (Coughlan et al., 2019; Puthusseryppady et al., 2022), *navigation test suite* (Laczó et al., 2021, 2022), *virtual supermarket test* (Coughlan et al., 2020; Puthusseryppady et al., 2022), and *object location task* (Castegnaro et al., 2022) provide starting points to build unified paradigms and tools for the development of scalable diagnostic tools and should be considered to new analyses of evidence in future reviews.

### Implications for Research

The critical analysis of the existing evidence available sheds light on the future directions for designing and enhancing VRSG-based instruments in the context of early AD detection. Further research is required to establish the psychometric performance and clinical utility of VRSG-based instruments, particularly the content validity, usability, and diagnostic accuracy for preclinical AD. Some of the identified VRSG-based instruments using path integration and wayfinding tasks have demonstrated adequate diagnostic accuracy to discriminate healthy controls, early-stage symptomatic AD, and early dementia due to AD (Allison et al., 2016, 2019; Castegnaro et al., 2022; Laczó et al., 2022; Levine et al., 2020). New research should be focused on testing the criterion validity to discriminate between subjects in preclinical stages of AD and MCI. To enhance the comparability of results, we encourage using the National Institute on Aging and Alzheimer’s Association research framework (Jack et al., 2018) as the standard of reference for preclinical AD identification. Other scenarios can include testing VRSG-based instruments in populations with genetic risk factors for sporadic and familial AD (Fuller et al., 2019; Sepulveda-Falla et al., 2012). The innovative approach of utilizing a widely accessible video game, such as *Sea Hero Quest* (Coughlan et al., 2018; Coutrot et al., 2018), has the potential to facilitate the enrollment of participants.

Regarding the theoretical framework supporting VRSG-based instruments, our findings establish the cognitive map hypothesis (O’Keefee & Nadel, 1978; Tolman, 1948) as the foundational theory in designing VRSG-based instruments and virtual reality paradigms for AD research. This hypothesis is supported by empirical evidence, such as the observed hippocampal neural response during virtual navigation (Epstein et al., 2017). Additionally, other variables related to spatial cognition and general cognitive status could be considered as proxy variables for determining cognitive domain and clinical utility, respectively (Burgess et al., 2004; Hirtle, 2013; Vasilyeva, 2005).

Identified VRSG-based instruments have limitations in providing evidence about the predictive value of the diagnosis and the impact of diagnostic testing on the progression and management of the disease (Knottnerus & Buntinx, 2009; Pepe, 2003). Therefore, new research, including cohort studies, should be considered to determine the operative characteristics of the instruments. Notably, the reliance on common statistics like Cronbach’s alpha for reliability assessment is increasingly viewed as insufficient (Sijtsma, 2009). In the context of AD, a degenerative condition with varying cognitive decline time rates (Morrison et al., 2015; Potashman et al., 2023), longitudinal studies are particularly valuable. They can provide deeper insights into the test–retest reliability and responsiveness of VRSG-based instruments (Coughlan et al., 2020; Mokkink et al., 2018), elements crucial for tracking progression of the disease. Given the expected cognitive changes, it is essential to provide a rationale for selecting the recall period to examine potential learning effects and clinical changes associated with disease progression. Following the recommendations for study design suggested by COSMIN (Mokkink et al., 2019) and the considerations for diagnostic research (Knottnerus & Buntinx, 2009; Pepe, 2003) can be helpful to enhance the comparability of results.

In summary, this review underscores the potential of VRSG-based instruments for early AD detection, while also identifying gaps in evidence and critical areas for future research. These instruments, in their current state, stand out as a promising source of cognitive biomarkers for detecting AD from its preclinical stage. However, heterogeneity in research designs and the risk of bias across studies limit their clinical application. Overcoming these challenges calls for joint efforts from clinicians and researchers, advancing these technologies as practical diagnostic tools on a larger scale. For researchers developing VRSG-based instruments, we advise integrating a theoretical framework anchored in neuroscience. The cognitive map hypothesis is particularly suitable, due to its neurological underpinnings and its degree of support within the current knowledge base. Moreover, we strongly recommend that researchers also clearly articulate and rationalize specific hypotheses for construct validity testing, adhere to the NIAA’s research framework for AD diagnosis as the most reliable standard, and assess usability through data collection on digital ergonomics and user interface via qualitative methods, such as interviews, focus groups, or questionnaires (Ben-sadoun et al., 2018; Lewis, 2018; Sauro & Lewis, 2012). Crucially, publishing evidence on validity and usability from early development stages, including pilot studies involving individuals across the AD continuum, will significantly contribute to instrument refinement and effectiveness.

## Limitations

To the best of our knowledge, this study represents a pioneering effort in using standardized criteria to analyze the quality of studies on measurement properties for VRSG-based instruments. Nonetheless, some limitations should be recognized. Our adherence to the COSMIN guidelines, which are tailored for PROMs, primarily resulted in a focused yet somewhat restricted scope of evaluation. Notably, the COSMIN framework does not explicitly address aspects such as instrument usability and norming, which are critical for analyzing VRSG-based instruments. Additionally, while ten studies reported pilot studies (Allison et al., 2019; Caffò et al., 2012; Davis & Sikorskii, 2020; Laczó et al., 2022; Levine et al., 2020; Parizkova et al., 2018; Puthusseryppady et al., 2022; Ritchie et al., 2018; Tarnanas et al., 2012; Zen et al., 2013) and one study reported the assessment of applicability (Silva et al., 2023), the lack of standardized criteria for their evaluation limited our analysis. This constraint impeded a thorough assessment of factors influencing instrument usage, such as age and technological literacy. Consequently, our study could not provide an exhaustive set of recommendations for best practices in evaluating these essential aspects of VRSG-based instruments, underlining the need for further research and development of comprehensive assessment criteria in this field.

Our examination focused on studies published from 2012 onwards, across eight major databases. Consequently, investigations reporting on the psychometric properties of VRSG-based instruments published before 2012 are beyond the scope of this work. Although a gray literature search was conducted, it is possible that other sources of evidence still need to be explored. For instance, personal notes and non-published works may contain valuable insights regarding developments, potential paradigms, or pilot studies that could contribute to advancing and refining the VRSG-based instruments. We encourage researchers to publish pilot studies, proof-of-concept, and early designs to advance the standardization and development of virtual reality as feasible technology for human spatial navigation assessment.

The inclusion criteria for this review, which primarily targeted references in English and Spanish, introduce a potential language bias (Pieper & Puljak, 2021). Although English is the predominant language in scientific research (Meneghini & Packer, 2007) and databases such as SciELO, RedALyC, and LILACS provide access to literature in various languages, limiting our research to only English or Spanish may have inadvertently excluded other contributions. This is particularly true for non-peer-reviewed works published in other languages. This limitation underscores the need for more inclusive linguistic approaches in future research, particularly concerning VRSG-based instruments. Adopting such approaches would ensure a more comprehensive and globally diverse understanding of the subject.

Finally, it is important to note the limitations arising from the heterogeneous selection of reference standards in the included studies. This diversity in diagnostic criteria can affect the comparability of diagnostic accuracy and known-group validity, as different tools may yield varying results depending on the applied standards (Bossuyt et al., 2003). Recognizing this limitation underscores the need for standardized diagnostic criteria in future research, particularly when assessing the sensitivity of spatial navigation paradigms in Alzheimer’s disease and MCI.

## Conclusion

This systematic review has identified and evaluated 30 VRSG-based instruments, underscoring their potential for assessing spatial navigation and memory impairments from the earliest stages of AD. These instruments are of great clinical importance given that such deficits are among the initial signs of cognitive decline in at-risk populations (Castegnaro et al., 2022; Colombo et al., 2017; Nedelska et al., 2012). While these tools show promise in differentiating between symptomatic stages of AD—including MCI and dementia—from clinically normal individuals, their efficacy in preclinical stages remains to be fully established. Expansion of studies to include familial early-onset AD populations could provide crucial insights into AD pathology, independent of age-related factors, thereby enhancing population-level screening programs.

Our evaluation, as summarized in Table 3, indicates gaps in knowledge and potential biases in existing VRSG-based instruments. These instruments have generally demonstrated construct validity, comparing well-known groups in AD and correlating the performance in spatial navigation and spatial memory tasks with established visuospatial constructs and neural correlates. However, improvements in sampling strategies and statistical hypothesis formulation are needed to strengthen methodological robustness. Criterion validity is particularly challenged by the heterogeneity in standard reference selection, which can be addressed by adhering to contemporary AD research frameworks like that proposed by NIA-AA (Jack et al., 2018) or by providing comprehensive rationales for selecting at-risk groups. Furthermore, the adoption of unified theoretical frameworks will benefit the development of measurement models aligned with spatial navigation theories, thus enhancing structural validity assessments and contributing to the unification of spatial navigation models.

Longitudinal study designs are essential for establishing the reliability of the measures provided by VRSG-based instruments over time. The selection of follow-up times must be carefully rationalized to ascertain the reliability of an instrument. Improving the reporting of methodological details in studies is crucial for reducing risk of bias and enhancing the understanding of study designs (Mokkink et al., 2019). Therefore, researchers should adhere to guidelines like STARD for reporting diagnostic accuracy in dementia research (Noel-Storr et al., 2014), ensuring clarity in hypotheses, appropriate sampling methods, and adequate sample sizes for statistical power.

In conclusion, effectively addressing these identified gaps will significantly improve decision-making processes regarding the adoption of VRSG-based instruments as diagnostic tools for AD. By adhering to the study recommendations (outlined in supplementary material), researchers have an opportunity to substantially contribute to the evolution and refinement of VRSG-based instruments, advancing diagnostics in both the clinical and research domains of AD.

## Supplementary Information

Below is the link to the electronic supplementary material.

Supplementary file1 (DOCX 174 KB)

Supplementary file2 (DOCX 232 KB)

Supplementary file3 (DOCX 16 KB)

Supplementary file4 (XLSX 396 KB)

## Data Availability

The datasets and tables generated during the review are available in Open Science Framework repository, https://osf.io/fyjx4/?view_only=34c89a393d294ec29298c836b8c4aee4.
