# Supplementary material for: Virtual Reality and Serious Videogame-Based Instruments for Assessing Spatial Navigation in Alzheimer’s Disease: A Systematic Review of Psychometric Properties
Source: Neuropsychol Rev. 2024 Feb 26;35(1):77–101. doi: 10.1007/s11065-024-09633-7 (PMC11965194; doi:10.1007/s11065-024-09633-7)
Supplement: Supplementary file 1 — Supplementary file1 (DOCX 174 KB) [file 11065_2024_9633_MOESM1_ESM.docx]

**Supplementary Material.**

**Supplementary Table 1.** Databases and search strategy

| Database | Search Strategy. |
| --- | --- |
| Scopus  PUBMED/Medline  EBSCO  Web of Science  PROQUEST  HMIC | (“Spatial Navigation” OR “Spatial Memory”) AND ("Alzheimer*" OR "Alzheimer Disease" OR "Mild Cognitive Impairment" OR "Dementia") AND ("Serious Game*" OR "Virtual Reality" OR "Virtual" OR "Maze Learning" OR "Task") NOT (train* OR rehabilitat* OR drug* OR "Parkinson Disease" OR "Vascular Dementia" OR "VIH" OR "Diabetes" OR "Mice" OR "Rat" OR "Dog”) |
| PsycNET (PsycArticles, PsycINFO, PsycEXTRA) | **Any Field:** “Spatial Navigation” OR “Spatial Memory” AND **Any Field:** "Alzheimer*" OR **Any Field:** "Alzheimer Disease" OR **Any Field:** "Mild Cognitive Impairment" OR **Any Field:** "Dementia" AND **Any Field:** "Serious Game*" OR **Any Field:** "Virtual Reality" OR **Any Field:** "Virtual" OR **Any Field:** "Maze Learning" OR **Any Field:** "Task" NOT **Any Field:** train* OR rehabilitat* OR drug* OR "Parkinson Disease" OR "Vascular Dementia" OR "VIH" OR "Diabetes" OR "Mice" OR "Rat" OR "Dog” AND **Population Group:** Human |
| Google Scholar | “Spatial Navigation” OR “Spatial Memory” +"Alzheimer Disease" OR “Mild Cognitive Impairment” -rat -mice -diabetes –Parkinson -training -VIH -rehabilitation |
| SciELO | ("espacial") AND ("Alzheimer" OR "deterioro cognitivo leve") AND ("virtual" OR "juego serio") |
| RedALyC | (“espacial”) AND (“realidad virtual” OR “juegos serios”) AND (“Alzheimer” OR “deterioro cognitivo leve”) |
| BVS (LILACS  WHO IRIS  Index Psicología – Periódicos  SciELO Preprints) | (“navegación espacial” OR “memoria espacial”) AND (“alzheimer” OR “enfermedad de alzheimer” OR “deterioro cognitivo leve” OR “deterioro cognitivo subjetivo” OR “demencia”) |

**Supplementary Table 2.** Search strategies for Open Science Framework

| Equation | Search term | Number of results |
| --- | --- | --- |
| #1 | “Alzheimer’s disease” | 73 |
| #2 | “Alzheimer” | 30 |
| #3 | “Alzheimer’s” | 86 |
| #4 | “Mild cognitive impairment” | 63 |
| #2 | “spatial navigation” | 51 |
| #6 | “spatial memory” | 42 |
| #7 | “spatial” | 2420 |
| #3 | “virtual reality” | 454 |
| #9 | “serious games” | 27 |
| Search equation 1 | #3 AND #9 | 2 |
| Search equation 2 | #4 AND #9 | 1 |
| Search equation 3 | #3 AND #9 | 2 |
| Search equation 4 | #3 AND #6 | 4 |

**Note:** Search equation 4 was selected for the systematic review due to the relevance of their findings.

**Supplementary Material.** Search strategy for Latin-American repositories description

A preliminary search across 46 university repositories was conducted, focusing on graduate programs in neuroscience and related fields, as listed in the *Sistema Nacional de Información de la Educación Superior* (SNIES). Despite identifying 186 theses, none met our eligibility criteria.

The search strategy varied across repositories due to differences in their search engines. We utilized a combination of terms from Table 3, employing advanced search strategies where available. In cases with more basic search functionalities, we used keywords related to Alzheimer's Disease and spatial navigation. For inclusivity, we used these search terms in Latin-American databases. This ensured accurate translations and effective application in searching for relevant academic works in neuropsychology and related programs across various repositories in the region.

**Supplementary Table 3.** Search equation and terms used in searching for thesis and dissertation finding in Colombian Universities.

| Colombian universities repositories for Thesis and dissertations | “Alzheimer” AND “Navegación especial”  “Alzheimer” AND “navegación”  “Deterioro cognitivo leve” AND “Navegación espacial” |
| --- | --- |
|  | “Alzheimer”  “Memoria espacial”  “Realidad virtual”  “Navegación espacial” |
|  | (“Navegación Espacial” OR “Memoria Espacial”) AND (“Alzheimer” OR “Enfermedad de Alzheimer” OR “Deterioro Cognitivo Leve” OR “Deterioro Cognitivo Subjetivo” OR “Demencia”) AND (“Juego serio” OR “Realidad virtual” OR “Laberinto” OR “Tarea” OR “Software”) |

**Supplementary Table 4.** VRSG-based instruments description

| **Reference** | **VR-SG-Based Instrument** | **Description** | **Construct(s)** | **Target Population** |
| --- | --- | --- | --- | --- |
| Puthusseryppady et al., (2022) Coughlan et al., (2020) Coughlan et al., (2019) Gellersen et al., (2021) | Sea Hero Quest (SHQ). | It is a mobile game that assesses spatial navigation abilities in laboratory and online settings. The game involves navigating a boat to various locations in a VR ocean environment on an iPad, with two levels: wayfinding and flare. Wayfinding levels require participants to use a cognitive map to navigate to numbered checkpoints, and the outcome variables are total distance and duration. Flare levels require participants to navigate to a flare gun and shoot it toward the starting location. | Spatial navigation, Spatial memory. | AD. |
| Da Costa et al., (2022)  Da Silva et al., (2023) | SOIVET (Maze Task and Route Task). | A VR test designed to assess spatial orientation abilities. It consists of two tasks - SOIVET-Maze and SOIVET-Route - which focus on allocentric to egocentric spatial ability and visuospatial memory, respectively. The test was adapted for the Samsung Gear VR™ immersive interaction system and includes automatically recorded and extractable data. Preliminary results indicate good tolerability, sense of presence, and usability. | Spatial orientation. | MCI or AD. |
| Ritchie et al., (2018) | Reality Supermarket Trolley Task. | Involves watching 14 short video clips within a simulated supermarket. Participants view the videos from a first-person perspective and must follow a route through the supermarket, which includes a series of 90-degree turns, after which they must point toward the entrance. The response is recorded according to a theoretical quadrant, and the number of correct locations and error types is noted. The total score range for the VRST is 0-14. | Spatial processing. | AD. |
| Coughlan et al., (2020) Puthusseryppady et al., (2022) | Virtual supermarket test (VST). | It is a short test that measures path integration through four sub-tests: egocentric orientation, heading direction, allocentric memory, and central navigation preference. The test has two alternative forms, a paper-based response version and an electronic version. Both versions have 14 identical trials. | Spatial memory. | APOE carriers. |
| Kunz et al., (2015) | Object location memory task. | It consists of a circular virtual arena. At the beginning of each trial, a cue is presented, which had to be placed as accurately as possible at its correct location during the next task phase. Participants received feedback after each trial. The averaged distance between response locations and correct across trials is calculated as a performance measure. | Object location memory. | AD. |
| Parizkova et al., (2018) | Y-Maze strategy assessment (yVSA). | The original yVSA instructions were adapted for cognitively impaired participants. The task consists of five blocks, each containing training and probe trials, to determine stable strategy preference moving through a virtual environment using a joystick. The trials were designed to assess allocentric vs egocentric spatial strategies preferences. | Spatial navigation (Allo and Ego strategies). | aMCI and AD. |
| Allison et al., (2019) | Modified cognitive mapping task. | It is a modified version of a maze task originally published in Allison et al. (2016). It involves a non-immersive virtual maze environment created in WorldViz Vizard and presented on a desktop computer. The environment consists of interconnected hallways with 18 ambient cues and four wallpaper patterns of different colors. | Allo strategy, Relational binding, Visual perspective. | Preclinical AD. |
| Morganti et al., (2013) | VR-Maze spatial task. | The task assesses the allocation of spatial knowledge in participants by asking them to draw the path from start to exit in one of five complex mazes. Then, participants are asked to locate the exit point in the corresponding virtual reality version of the maze. The start and exit points are indicated in both versions, and the sun helps orient the participant during the exploration. | Egocentric and allocentric spatial strategies. | AD. |
| Morganti et al., (2013) | VR-Road Map Task. | It is a spatial navigation test that involves both paper-and-pencil and virtual reality components. Participants must first complete the Money's Road Map Test, which requires them to indicate the direction taken at each turn to follow a designated route on a stylized city map. Next, they navigate the corresponding virtual reality version of the map, actively deciding which direction to turn at each intersection. The test assesses the allo-to egocentric translation of spatial knowledge required for navigation in a virtual environment. | Egocentric and allocentric spatial strategies. | AD. |
| Castegnaro et al., (2022) | The Object Location Task. | It is conducted using the HTC Vive iVR kit, which includes a 4.0 x 4.0 meter tracked walkable area. The task, developed with the Unity game engine, consists of three subtasks: (i) spatial memory, where participants learn the locations of objects in a cue-rich environment and replace them after a short delay, (ii) object recognition, where participants indicate whether a cued object was previously seen, and (iii) context memory, where participants link previously seen objects to the environment they belong to. The task includes visually distinct environments created using different textures and landmarks to aid object-context binding. | Object location memory. | AD. |
| Laczó et al., (2022) Laczo et al., (2021) | Navigation Test Suite. | It is a set of three virtual spatial tasks designed to evaluate wayfinding and route learning abilities in a realistic virtual city. The tasks include route repetition, route retracing, and directional approach, which requires participants to recall the street from which they originally approached an intersection when approaching it from a viewpoint they had not experienced before. | Spatial navigation. | AD. |
| Lesk et al., (2014) | Virtual Reality for early detection of AD (VREAD). | It consists of three modules, VR Practice, VR Park, and VR Games, intending to test the participants' spatial navigation abilities. The participants navigate different levels by following the red lines attached to a path to reach a target destination. The task records and analyzes the participants' performance. Finally, the participant's ability to recall the given path is tested. | Topographic memory. | MCI or AD. |
| Caffò et al., (2012)  Caffò et al., (2018) | Virtual Reorientation Test (VReoT). | It is a tool to assess reorientation performance in individuals with cognitive impairment. It consists of 5 subtests, each with a different combination of spatial information. These include layout only, layout and positional landmark, layout and directional landmark, positional landmark, and directional landmark only. | Spatial reorientation. | MCI. |
| Lee et al., (2014) | VRAM Task. | It is an analogue of the radial arm maze test used in small animals like rodents. The VRAM allows for the simultaneous examination of spatial working memory and reference memory using a computer-human interface. Participants use monitors and joysticks to navigate through the virtual reality environment. | Spatial working memory. Spatial reference memory. | aMCI and AD. |
| Tarnanas et al., (2015) | Complex Activities of Daily Living. | This serious game includes two modules: DOT and NAV. DOT is a virtual apartment building fire evacuation drill consisting of six scenarios, while NAV is a navigation task with six levels of increasing difficulty. Both tasks required medium to high cognitive control processes, such as inhibition of external stimuli and processing speed. | Spatial navigation. | aMCI and AD. |
| Levine et al., (2020) Allison et al., (2016) | Cognitive Mapping Task. | It consists of learning and a retrieval phase. During the learning phase, participants explored an environment and placed Xs on a 2D map at landmark locations. The retrieval phase occurred after a delay, where participants found landmarks presented in a picture using the shortest path as quickly as possible. Dependent variables included time travelled, landmark recall, landmark identification, and scene recognition, with scores standardized to obtain an individual's relative ranking and the same scale across studies. | Spatial navigation. | AD. |
| Levine et al., (2020) | Route Learning Task. | The task involves learning and delayed retrieval phases. Participants follow a marked route repeatedly for 5 minutes and draw the path on a blank map. The learning score is the average proportion of correctly drawn turns at intersections. After a 10-minute delay, participants traversed the designated route without arrows three times, and the average time taken to traverse the route is the retrieval phase variable score. | Spatial navigation. | AD. |
| Migo et al., (2016) | Platform Task. | The task requires participants to navigate a circular arena with visual cues and select each circular platform without revisiting any. The task uses allocentric strategies, and feedback is given after each selection. The task is self-paced, and the difficulty varies by changing the number of platforms. | Spatial navigation. | aMCI. |
| Tarnanas et al., (2012) | Virtual Action Planning Museum (VAP-M). | It is a 3D virtual reality cognitive screening tool that includes tasks for route planning, verbal episodic memory, and visuospatial working memory. The tasks are similar to those used in a museum visit and reflect current process-specific assessments. The virtual environment is modeled after the Archeological Museum of Aiani in Greece, and the task is designed to detect cognitive abnormalities in older adults. | Spatial cognition. | aMCI. |
| Konishi et al., (2018) | Concurrent Spatial Discrimination Task (CSDLT). | This task was created using Unreal Engine, the task was adapted from a radial maze model that involves spatial discrimination learning in rodents. The test consists of a central platform with 12 corridors branching off from it. An enriched environment surrounds the labyrinth with mountains, trees, and other landmarks. | Spatial memory. | MCI or AD. |
| Bellassen et al., (2012) | The Starmaze task. | This task is a 3D virtual reality environment designed and developed using 3D StudioMax and made interactive with Virtools. It consists of five central alleys forming a pentagon, with five alleys branching off from the angles of the pentagon. Participants use a joystick to move their point of view forward or backward or to turn left or right. Distant environmental landmarks surround the pentagon. The task includes training trials followed by tests of spatial and temporal memory. | Spatiotemporal memories. | Preclinical AD. |
| Park et al., (2022) | Spatial Cognitive Task Based on Virtual Reality (SCT-VR). | It is an immersive virtual environment in which participants use a joystick to navigate through an open arena with boundary cues. Participants start at a randomly selected location and are instructed to find and collect a gem at a specific location before being presented with another gem to collect. After collecting the second gem, participants are instructed to navigate back to their starting location and confirm their location by pressing a button. | Spatial memory. | MCI. |
| Bayahya et al., (2021) | The virtual scenario. | The task starts with an automatic tour of a street overlooking the sea with shops and a supermarket. The participant completes two cognitive tests: visuospatial function and memory function, with specific scenarios for each task. The system compiles data and calculates cognitive scores based on the performance in each test. | Navigational processes. | AD. |
| Serino et al., (2015) Serino et al., (2018) | Virtual Room Environment. | The task involves a virtual room with two objects, a plant and a stone, and an arrow indicating the North. Participants are instructed to memorize the position of the plant in three different trials. In the retrieval phase, participants complete two tasks: Task 1 requires participants to indicate the position of the plant on a real map. In contrast, Task 2 requires participants to indicate the position of the plant in an empty virtual room using allocentric viewpoint-independent and viewpoint-dependent representations. The accuracy of spatial location is the dependent variable. The task was created using NeuroVirtual 3D, an extension of NeuroVR for neuropsychological assessment and neurorehabilitation. | Spatial navigation: Egocentric and allocentric spatial strategies. | MCI or AD. |
| Mohammadi et al., (2018) | Virtual Neighbourhood task (VRNT). | The task includes two virtual environments: a virtual neighbourhood and a virtual maze. The virtual neighbourhood contains navigational landmarks to aid participants in finding their way, while the virtual maze does not have any landmarks. Participants are shown a 2D overhead view of the environment for 60 seconds, and then a 3D first-person view is presented. Participants are instructed to find a specified goal and have three familiarization trials and five assessment trials. Correct and incorrect responses and completion times are recorded for each trial. | Egocentric and allocentric spatial strategies. | MCI and AD. |
| Zen et al., (2013) Moussavi et al., (2022) | Virtual Reality Navigational experiment (VRN). | It is a virtual reality navigation task where participants must find a target room marked with an "X" in a virtual cubic three-story building. The building looks identical on each side and has eight rooms on each floor with stairs in the middle. Participants are shown the location of the target room after the building is rotated and then must find the room within a trial. There are eight trials, and if the participant is completely lost after three attempts, they can skip the trial. Positive feedback is given upon finding the target room. | Spatial perception. Spatial orientation. | AD. |
| Davis, R., & Sikorskii, A. (2020) | VR simulation of a large senior residence. | It is a VR simulation of a large senior retirement community projected on a 12-foot screen. The participants are requested to find their way, moving throughout the environment using a joystick. Each trial has a duration of 3 minutes. | Visual attention. | MCI or AD. |
| Bierbrauer et al., (2020)  Colmant, et.al., (2023) | The Apple Game | It is a task conducted in a simple virtual reality environment where participants navigate a grassy field under a blue sky to remember and locate objects. The task involved three phases: initially finding and memorizing a basket's location, then locating up to five trees in different places, each potentially holding an apple, and finally returning to the basket's location to 'place' an apple found on the last tree. Performance is assessed based on the accuracy of remembering the basket's location. The task varied the environment by including or excluding visual cues like walls or a lighthouse, to test different navigation skills. | Spatial orientation.  Path integration. | APOE carriers. |
| Pink et. al., (2023) | Virtual reality-based path integration task | The scenario featured a circular island with three distinct landmarks: a mountain, a raft, and a pirate ship. Participants engage in tasks to test their spatial and item memory. The spatial memory task had two conditions: continuous navigation and teleportation. In continuous navigation, subjects collect a target image and navigate to a new location, then to the target location. In teleportation, they are transported to a new location after image collection, then navigate to the target. Performance is measured by proximity to the target location, with rewards in virtual gold coins. The item memory task involved choosing between target and lure images, similar to each other, from a set of 143 image pairs, with correct choices also rewarded with coins. The experiment aimed to assess memory without reliance on landmarks, encouraging metric computation. | Spatial memory. | APOE carriers. |
| Plaza-Rosales, et.al., (2023) | Virtual Morris Water Navigation (VMWN) | In this task participants navigate a simulated Morris water maze. The environment features a circular pool in a room with visual cues on the walls. The room measures 16 units with a 3.2-unit diameter pool. Participants use keyboard buttons to find a hidden platform in the pool. The experiment comprises three stages: Training, Task I, and Task II. In Training, participants locate a hidden platform, which becomes visible after 1 minute if not found, across four trials. Task I involves finding the platform with different visual cues in 20 trials, divided into four blocks. Task II requires choosing the correct platform from two visible options in an equivalent room with different cues, also in 20 trials. The program records the navigational route for analysis. | Spatial navigation. | MCI. |

AD = Alzheimer Disease, MCI = Mild Cognitive Impairment, aMCI = Amnestic Mild Cognitive Impairment.

**References**

Allison, S. L., Fagan, A. M., Morris, J. C. & Head, D. (2016). Spatial Navigation in Preclinical Alzheimer’s Disease Samantha. *Journal of Alzheimer’s Disease*, *52*(1), 77–90. <https://doi.org/10.3233/JAD-150855>.

Allison, S. L., Rodebaugh, T. L., Johnston, C., Fagan, A. M., Morris, J. C. & Head, D. (2019). Developing a Spatial Navigation Screening Tool Sensitive to the Preclinical Alzheimer Disease Continuum. *Archives of Clinical Neuropsychology*, *34*(7), 1138–1155. <https://doi.org/10.1093/arclin/acz019>

Bayahya, A. Y., Alhalabi, W. & Alamri, S. H. (2021). Smart health system to detect dementia disorders using virtual reality. *Healthcare (Switzerland)*, *9*(7) 810. <https://doi.org/10.3390/healthcare9070810>

Bellassen, V., Iglói, K., de Souza, L. C., Dubois, B. & Rondi-Reig, L. (2012). Temporal order memory assessed during spatiotemporal navigation as a behavioral cognitive marker for differential Alzheimer’S disease diagnosis. *Journal of Neuroscience*, *32*(6), 1942–1952. <https://doi.org/10.1523/JNEUROSCI.4556-11.2012>

Bierbrauer, A., Kunz, L., Gomes, C. A., Luhmann, M., Deuker, L., Getzmann, S., Wascher, E., Gajewski, P. D., Hengstler, J. G., Fernandez-Alvarez, M., Atienza, M., Cammisuli, D. M., Bonatti, F., Pruneti, C., Percesepe, A., Bellaali, Y., Hanseeuw, B., Strange, B. A., Cantero, J. L. & Axmacher, N. (2020). Unmasking selective path integration deficits in Alzheimer’s disease risk carriers. *Science Advances, 6*(35). <https://doi.org/10.1126/sciadv.aba1394>

Caffò, A. O., De Caro, M. F., Picucci, L., Notarnicola, A., Settanni, A., Livrea, P., Lancioni, G. E. & Bosco, A. (2012). Reorientation deficits are associated with amnestic mild cognitive impairment. *American Journal of Alzheimer’s Disease and Other Dementias*, *27*(5), 321–330. <https://doi.org/10.1177/1533317512452035>

Caffò, A. O., Lopez, A., Spano, G., Serino, S., Cipresso, P., Stasolla, F., Savino, M., Lancioni, G. E., Riva, G. & Bosco, A. (2018). Spatial reorientation decline in aging: the combination of geometry and landmarks. *Aging and Mental Health, 22*(10), 1372–1383. <https://doi.org/10.1080/13607863.2017.1354973>

Castegnaro, A., Howett, D., Li, A., Harding, E., Chan, D., Burgess, N. & King, J. (2022). Assessing mild cognitive impairment using object-location memory in immersive virtual environments. *Hippocampus*, *32*(9), 660–678. <https://doi.org/10.1002/hipo.23458>

Colmant, L., Bierbrauer, A., Bellaali, Y., Kunz, L., Van Dongen, J., Sleegers, K., Axmacher, N., Lefèvre, P. & Hanseeuw, B. (2023). Dissociating effects of aging and genetic risk of sporadic Alzheimer’s disease on path integration. *Neurobiology of Aging, (131)*, 170–181. <https://doi.org/10.1016/j.neurobiolaging.2023.07.025>

Coughlan, G., Coutrot, A., Khondoker, M., Minihane, A. M., Spiers, H. & Hornberger, M. (2019). Toward personalized cognitive diagnostics of at-genetic-risk Alzheimer’s disease. *Proceedings of the National Academy of Sciences of the United States of America*, *116*(19), 9285–9292. <https://doi.org/10.1073/pnas.1901600116>

Coughlan, G., Puthusseryppady, V., Lowry, E., Gillings, R., Spiers, H., Minihane, A. M. & Hornberger, M. (2020). Test-retest reliability of spatial navigation in adults at-risk of Alzheimer’s disease. *PLoS ONE*, *15*(9), e0239077. <https://doi.org/10.1371/journal.pone.0239077>

Da Costa, R. Q. M., Pompeu, J. E., Moretto, E., Silva, J. M., Dos Santos, M. D., Nitrini, R. & Brucki, S. M. D. (2022). Two Immersive Virtual Reality Tasks for the Assessment of Spatial Orientation in Older Adults with and Without Cognitive Impairment: Concurrent Validity, Group Comparison, and Accuracy Results. *Journal of the International Neuropsychological Society*, *28*(5), 460–472. <https://doi.org/10.1017/S1355617721000655>

Davis, R. & Sikorskii, A. (2020). Eye Tracking Analysis of Visual Cues during Wayfinding in Early Stage Alzheimer’s Disease. *Dementia and Geriatric Cognitive Disorders*, *49*(1), 91–97. <https://doi.org/10.1159/000506859>

Gellersen, H. M., Coughlan, G., Hornberger, M. & Simons, J. S. (2021). Memory precision of object-location binding is unimpaired in APOE ϵ4-carriers with spatial navigation deficits. *Brain Communications*, *3*(2). <https://doi.org/10.1093/braincomms/fcab087>

Konishi, K., Joober, R., Poirier, J., MacDonald, K., Chakravarty, M., Patel, R., Breitner, J. & Bohbot, V. D. (2018). Healthy versus entorhinal cortical atrophy identification in asymptomatic APOE4 carriers at risk for Alzheimer’s disease. *Journal of Alzheimer’s Disease*, *61*(4), 1493–1507. <https://doi.org/10.3233/JAD-170540>

Kunz, L., Navarro Schröder, T., Lee, H., Montag, C., Lachmann, B., Sariyska, R., Reuter, M., Stirnberg, R., Stöcker, T., Messing-Floeter, P. C., Fell, J., Doeller, C. F. & Axmacher, N. (2015). Reduced grid-cell-like representations in adults at genetic risk for Alzheimer’s disease. *Science*, *350*(6259), 430–433. <https://doi.org/10.1126/science.aad1171>

Laczó, M., Martinkovic, L., Lerch, O., Wiener, J. M., Kalinova, J., Matuskova, V., Nedelska, Z., Vyhnalek, M., Hort, J. & Laczó, J. (2022). Different Profiles of Spatial Navigation Deficits In Alzheimer’s Disease Biomarker-Positive Versus Biomarker-Negative Older Adults With Amnestic Mild Cognitive Impairment. *Frontiers in Aging Neuroscience*, *14*. <https://doi.org/10.3389/fnagi.2022.886778>

Laczó, M., Wiener, J. M., Kalinova, J., Matuskova, V., Vyhnalek, M., Hort, J. & Laczó, J. (2021). *Spatial Navigation and Visuospatial Strategies in Typical and Atypical Aging*. <https://doi.org/10.3390/brainsci>

Lee, J., Kho, S., Yoo, H. Bin, Park, S. & Choi, J. (2014). Spatial memory impairments in amnestic mild cognitive impairment in a virtual radial arm maze. *Neuropsychiatric Disease and Treatment*, *10*, 653–660. <https://doi.org/10.2147/NDT.S58185>

Lesk, V. E., Wan Shamsuddin, S. N., Walters, E. R. & Ugail, H. (2014). Using a virtual environment to assess cognition in the elderly. *Virtual Reality*, *18*(4), 271–279. <https://doi.org/10.1007/s10055-014-0252-2>

Levine, T. F., Allison, S. L., Stojanovic, M., Fagan, A. M., Morris, J. C. & Head, D. (2020). Spatial navigation ability predicts progression of dementia symptomatology. *Alzheimer’s and Dementia*, *16*(3), 491–500. <https://doi.org/10.1002/alz.12031>

Migo, E. M., O’Daly, O., Mitterschiffthaler, M., Antonova, E., Dawson, G. R., Dourish, C. T., Craig, K. J., Simmons, A., Wilcock, G. K., McCulloch, E., Jackson, S. H. D., Kopelman, M. D., Williams, S. C. R. & Morris, R. G. (2016). Investigating virtual reality navigation in amnestic mild cognitive impairment using fMRI. *Neuropsychology, Development, and Cognition. Section B, Aging, Neuropsychology and Cognition*, *23*(2), 196–217. <https://doi.org/10.1080/13825585.2015.1073218>

Mohammadi, A., Kargar, M. & Hesami, E. (2018). Using virtual reality to distinguish subjects with multiple- but not single-domain amnestic mild cognitive impairment from normal elderly subjects. *Psychogeriatrics*, *18*(2), 132–142. <https://doi.org/10.1111/psyg.12301>

Morganti, F., Stefanini, S. & Riva, G. (2013). From allo- to egocentric spatial ability in early Alzheimer’s disease: A study with virtual reality spatial tasks. *Cognitive Neuroscience*, *4*(3–4), 171–180. <https://doi.org/10.1080/17588928.2013.854762>

Moussavi, Z., Kimura, K. & Lithgow, B. (2022). Egocentric spatial orientation differences between Alzheimer’s disease at early stages and mild cognitive impairment: a diagnostic aid. *Medical and Biological Engineering and Computing*, *60*(2), 501–509. <https://doi.org/10.1007/s11517-021-02478-9>

Parizkova, M., Lerch, O., Moffat, S. D., Andel, R., Mazancova, A. F., Nedelska, Z., Vyhnalek, M., Hort, J. & Laczó, J. (2018). The effect of Alzheimer’s disease on spatial navigation strategies. *Neurobiology of Aging*, *64*, 107–115. <https://doi.org/10.1016/j.neurobiolaging.2017.12.019>

Park, J. H. (2022). Can the Virtual Reality-Based Spatial Memory Test Better Discriminate Mild Cognitive Impairment than Neuropsychological Assessment? *International Journal of Environmental Research and Public Health*, *19*(16). <https://doi.org/10.3390/ijerph19169950>

Pink, D., Ilkel, E., Chandreswaran, V., Moser, D., Getzmann, S., Patrick, G., Axmacher, N. & Zhang, H. (2023). Modeling the impact of genotype, age, sex, and continuous navigation on pathway integration performance. *BioRxiv.* <https://doi.org/10.1101/2023.09.11.556925>

Plaza-Rosales, I., Brunetti, E., Montefusco-Siegmund, R., Madariaga, S., Hafelin, R., Ponce, D. P., Behrens, M. I., Maldonado, P. E., & Paula-Lima, A. (2023). Visual-spatial processing impairment in the occipital-frontal connectivity network at early stages of Alzheimer’s disease. *Frontiers in Aging Neuroscience, 15*. <https://doi.org/10.3389/fnagi.2023.1097577>

Puthusseryppady, V., Morrissey, S., Spiers, H., Patel, M. & Hornberger, M. (2022). Predicting real world spatial disorientation in Alzheimer’s disease patients using virtual reality navigation tests. *Scientific Reports*, *12*(1). <https://doi.org/10.1038/s41598-022-17634-w>

Ritchie, K., Carrière, I., Howett, D., Su, L., Hornberger, M., O’Brien, J. T., Ritchie, C. W. & Chan, D. (2018). Allocentric and egocentric spatial processing in middle-aged adults at high risk of late-onset Alzheimer’s disease: The PREVENT dementia study. *Journal of Alzheimer’s Disease*, *65*(3), 885–896. <https://doi.org/10.3233/JAD-180432>

Serino, S., Morganti, F., Di Stefano, F. & Riva, G. (2015). Detecting early egocentric and allocentric impairments deficits in Alzheimer’s disease: an experimental study with virtual reality. *Frontiers in Aging Neuroscience*, *7*, 1–10. <https://doi.org/10.3389/fnagi.2015.00088>

Serino, S., Morganti, F., Colombo, D. & Riva, G. (2018). The Contribution of Allocentric Impairments to the Cognitive Decline in Alzheimer’s Disease. *Lecture Notes of the Institute for Computer Sciences, Social-Informatics and Telecommunications Engineering, LNICST*, *253*, 84–91. <https://doi.org/10.1007/978-3-030-01093-5_11>

Silva, J. M. da, Santos, M. D. dos, Costa, R. Q. M. da, Moretto, E. G., Viveiro, L. A. P. de, Lopes, R. de D., Brucki, S. M. D. & Pompeu, J. E. (2023). Applicability of an immersive virtual reality system to assess egocentric orientation of older adults. *Arquivos de Neuro-Psiquiatria, 81*(01), 019–026. <https://doi.org/10.1055/s-0042-1759762>

Tarnanas, I., Laskaris, N. & Tsolaki, M. (2012). On the comparison of VR-responses, as performance measures in prospective memory, with auditory P300 responses in MCI detection. *Studies in health technology and informatics, 181,* 156–161.

Tarnanas, I., Papagiannopoulos, S., Kazis, D., Wiederhold, M., Widerhold, B., Vuillermot, S. & Tsolaki, M. (2015). Reliability of a novel serious game using dual-task gait profiles to early characterize aMCI. *Frontiers in Aging Neuroscience*, *7*. <https://doi.org/10.3389/fnagi.2015.00050>

Zen, D., Byagowi, A., Tere Garcia Campuzano, M., kelly, D., Lithgow, B. & Moussavi, Z. (2013). *The Perceived Orientation in People with and without Alzheimer´s*. <https://doi.org/10.1109/ner.2013.6695971>
