# Supplementary material for: Virtual Reality and Serious Videogame-Based Instruments for Assessing Spatial Navigation in Alzheimer’s Disease: A Systematic Review of Psychometric Properties
Source: Neuropsychol Rev. 2024 Feb 26;35(1):77–101. doi: 10.1007/s11065-024-09633-7 (PMC11965194; doi:10.1007/s11065-024-09633-7)
Supplement: Supplementary file 2 — Supplementary file2 (DOCX 232 KB) [file 11065_2024_9633_MOESM2_ESM.docx]

**Supplementary Material.**

**Convergent validity: Correlation between VRSG-based instrument and neuropsychological tests.**

| Study | VRSG-based instrument | Reference Test | Correlation coefficient | p-value |
| --- | --- | --- | --- | --- |
| Morganti et.al., (2013) | VR-Maze spatial task | Corsi's Span | 0,51 | <,001 |
|  |  | Corsi's Supraspan | 0,70 | <,001 |
|  |  | Manikin test | 0,75 | <,001 |
|  |  | TMT | 0,46 | <,004 |
|  |  | Corsi's Span | 0,52 | <,001 |
|  | VR-Road Map Task | MMSE | 0,37 | <,018 |
|  |  | Corsi's Supraspan | 0,33 | <,049 |
| Lesk et.al., (2014) | Virtual Reality for early detection of AD (VREAD) | CANTAB PAL 6 error score | -0,45 | <,02 |
|  |  | GNT | 0,43 | <,02 |
| Lee et.al., (2014) | VRAM Task – WM Errors | Corsi’s Spatial Span Forward | -0,17 | <,05 |
|  |  | Corsi’s Spatial Span Forward | -0,29 | <,05 |
|  |  | CFRT Copy | -0,36 | <,05 |
|  |  | CFRT Immediate recall | -0,37 | <,05 |
|  |  | CFRT Delayed Recall | -0,39 | <,05 |
|  | VRAM Task – RM Errors | Corsi’s Spatial Span Forward | -0,17 | <,05 |
|  |  | Corsi’s Spatial Span Forward | -0,33 | <,05 |
|  |  | CFRT Copy | -0,37 | <,05 |
|  |  | CFRT Immediate recall | -0,44 | <,05 |
|  |  | CFRT Delayed Recall | -0,45 | <,05 |
|  | VRAM Task – Time | Corsi’s Spatial Span Forward | -0,17 | <,05 |
|  |  | Corsi’s Spatial Span Forward | -0,25 | <,05 |
|  |  | CFRT Copy | -0,37 | <,05 |
|  |  | CFRT Immediate recall | -0,37 | <,05 |
|  |  | CFRT Delayed Recall | -0,42 | <,05 |
|  | VRAM Task – Distance | Corsi’s Spatial Span Forward | -0,16 | <,05 |
|  |  | Corsi’s Spatial Span Forward | -0,31 | <,05 |
|  |  | CFRT Copy | -0,40 | <,05 |
|  |  | CFRT Immediate recall | -0,41 | <,05 |
|  |  | CFRT Delayed Recall | -0,41 | <,05 |
| Da Costa et.al., (2022) | SOIVET (Maze Task) | Money Road-Map test | 0,35 | <,05 |
|  |  | Tower of London | 0,35 | <,05 |
|  |  | BJLO | 0,43 | <,01 |
|  |  | ACE-R Total Score | 0,37 | <,05 |
|  |  | ACE-R Memory | 0,48 | <,01 |
|  |  | ACE-R Visuospatial | 0,32 | <,05 |
|  | SOIVET (Route Task) | MRMT | 0,39 | <,01 |
|  |  | ACE-R Total Score | 0,40 | <,05 |
|  |  | ACE-R Memory | 0,34 | <,05 |
|  |  | ACE-R Visuospatial | 0,32 | <,05 |

**Note:** TMT = Trial Making Test; MMSE = Minimental State Examination; ACE-R = Addenbrooke’s Cognitive Examination Revised; BJLO = Benton’s Judgment of Line Orientation Test; CFRT = Complex Figure Rey Test.

**Construct validity: Well-known groups comparison.**

| **Reference** | **VRSG-based instrument** | **Measure** | **Results** | **Observations** |
| --- | --- | --- | --- | --- |
|  |  |  |  |  |
| Bellassen et.al., (2012) | The Starmaze task | Sequential navigation | F(3,61) =13.3; *p*<10^-4^ | The authors used ANOVA tests to compare performances between groups. Sequential navigation performance in aMCI and AD participants were significantly impaired relative to controls, whereas AD participants’ performance was impaired relative to healthy controls in route tracing |
|  |  | Route tracing | F(3,61) =12.8; *p*<10^-4^ |  |
| Caffò et.al., (2012) | VReoT | Layout only condition | F_(2,101)_ = 9.47; *p*< .05; η_p_^2^ = 0.16 | One-way ANOVA was used to compare means from the three diagnostic groups for task score. Groups of aMCI participants performed worse than HC |
|  |  | Layout + landmark/target relationship | F_(2,101)_ = 33.74; *p*<.01; η_p_^2^ = 0.40 |  |
| Tarnanas et.al., (2012) | VAP-M | Spatial allocentric memory assessment | t_(25)_ = 2.057; *p* = .03 | Independent t-tests were used for assessing differences in the outcome measures. The aMCI participants’ performance was inferior to healthy controls. No effect size measure was reported |
|  |  |  |  |  |
| Morganti et.al., (2013) | VR-MST | Correct execution of each maze | F(89,22) = 89.22; *p* <.001; η_p_^2^ = 0.64 | Repeated measures ANOVA with Bonferroni's adjustment were used for comparison. The control group outperformed the AD group in the task |
| Morganti et.al., (2013) | VR-RMT | Correct answer (good direction at each turn) | F(9, 29); *p* < .004 | The AD group reached significantly fewer target points than the control group in the task |
| Zen et.al., (2013) | Virtual Reality Navigational experiment | Error score | *p*=2.5x10^-5^ | Mann Whitney U-test was used to contrast the performance between groups. AD participants performed worse than HC participants. No effect size or assumptions testing was reported |
| Lee et.al., (2014) | VRAM Task | Working memory errors | F=12.0; df=2; *p*<0.05; η_p_^2^=0.30 | Repeated measures ANOVAs revealed a significant effect of group on working memory. The HC and aMCI groups committed less errors than AD participants |
|  |  | Reference memory errors | F=17.7; df=2; *p*<0.05; η_p_^2^=0.38 |  |
| Lesk et.al., (2014) | VREAD | Movement measure by level | t_(29)_=3.17; *p*<0.01 | Independent t-tests were used for comparison. Level four score was lower in the MCI group compared to HC participants. No effect size was reported |
|  |  |  |  |  |
| Serino et.al., (2015) | Virtual Room Environment | Accuracy for spatial location | F(4,82) = 4.40; *p*<.01, η_p_^2^=0.18 | Repeated measures ANOVA with group (between-subject factor) and trials (within-subject factor). A significant effect of the interaction of Trials x groups was found. MCI participants perform worse in the third trial compared to HC and AD groups |
| Tarnanas et.al., (2015) | CADL | Accuracy-based IIV score | F_(2,225)_ = 5.75; *p* = 0.001; η^2^ = 0.06 | Univariate ANCOVA was used to evaluate group differences. The AD participants showed a higher across-domain IIV than both aMCI group and healthy control group |
|  |  | Latency-based IIV score | F_(2,225)_ = 5.71; *p* = 0.001; η^2^ = 0.06 |  |
| Migo et.al., (2016) | Platform task | Errors per trial | F(1,16) = 1.47, p = .243, f = .302 | Two-way repeated measures ANOVA with group (between-subject factor) and number of platforms (within-subject factor) showed a trend for an interaction between platform choice and group due to the aMCI group being slower than healthy controls |
|  |  |  |  |  |
|  |  | Group x platform choice | F(3,45) = 2.50, p = .072, f = .408 |  |
|  |  |  |  |  |
| Caffò et.al., (2018) | VReoT | Number of repetitions of the learning phase | F_(2,281)_ = 41.20; *p*< .001; η_p_^2^ = 0.22 | A one-way ANCOVA with group as between-subject variable was performed on the subtask involving only geometric information. MCI and probable dementia groups required a significantly higher number of trials than HC |
| Konishi et.al., (2018) | CSDLT | Navigational strategy (spatial vs response) | χ^2^= 0.05; *p* = .82 | No differences in the proportion of individuals who use spatial and response strategies between APOE ε4 carriers and non-carriers was found. Grey matter in the EC predicted navigational strategy with 92% accuracy |
| Mohammadi et.al., (2018) | VRNT | VN-Mean correct response | *p* < .001 | One-way ANOVA was used to evaluate the group differences between groups. AD participants performed significantly worse than MCI and HC participants in all tasks. No effect size measure was reported |
|  |  | VN-Mean response time | *p* < .001 |  |
|  |  | VM-Mean correct response | *p* < .001 |  |
|  |  | VM-Mean response time | *p* < .002 |  |
| Parizkova et.al. (2018) | yVSA | Allocentric navigation distance error | F(2) = 21.35; *p* < .001; η_p_^2^ = 0.49 | Authors performed a 3 (group) x 2 (gender) x 2 (strategy preference) ANOVA to compare the performance among the participants. Control group outperformed MCI and dementia groups (p < 0.001). Dementia group had worse performance than the aMCI group (p < 0.001) |
| Ritchie et.al., (2018) | Reality Supermarket Trolley Task | Supermarket test | *p* = .47 | Unpaired Wilcoxon test was used to compare the differences in the performance between participants with and without familial history of AD. No differences were reported |
|  |  |  |  |  |
| Serino et.al., (2018) | VR-Based procedure | Allocentric abilities | t_(28)_=-2,74; *p*=0.01; *d*=1.00 | Independent t-tests were used for assessing differences in the allocentric abilities. Cohen's d values over 0,8 were considered as high effect size by authors |
| Bierbrauer et al., (2020) | The Apple Game | Pure path integration | z ≥ 2.44, P_Tukey_ ≤ 0.039 | Mixed linear models were used to determine the effects of genotype and covariates (age, sex) in path integration performance. Controls outperformed risk carriers in pure path integration subtask. An interaction between APOE and incoming distance was observed, presumably reflecting stronger error accumulation in risk carriers than controls. The performance of risk carriers declined more strongly with increasing goal-to-landmark distance than the performance of controls. |
|  |  | Incoming distance | F = 3.94, p = 0.047 |  |
|  |  | Goal-to-landmark distance | F = 4.55, p = 0.033 |  |
| Coughlan et.al., (2019) | Sea Hero Quest | Wayfinding distance | b = 0.22; *p* = 0.004 | Mixed-effects models, with subject-level random effects, adjusted for age, sex, and baseline cognitive ability were used for comparison. The ε3ε3 carriers traveled a shorter distance during the task than ε3ε4 carriers |
| Davis & Sikorskii (2020) | VR simulation of a large senior residence | Percentage of fixations during wayfinding | F(1,13) = 6.79; *p*=.02 | Linear mixed-effect models were used for analyzing the percentage and the duration of fixations between groups. The AD group had a significantly lower percentage and duration of fixations after controlling for sex |
|  |  | Duration of fixations during wayfinding | F(1,13) = 4.87; *p*=.04 |  |
| Bayahya et.al., (2021) | The virtual scenario | Visuospatial function | *p* < .001 | Kruskall-Wallis H-test was used for analyzing the differences in participants’ group performance. AD participants performed worse than MCI participants in all measures. In turn, MCI participants performed worse than healthy controls on all tasks. No statistics values or effect size measures were reported |
|  |  | Visual perception and orientation | *p* < .001 |  |
|  |  | Visual memory | *p* < .001 |  |
| Gellersen et.al., (2021) | Sea Hero Quest | Wayfinding distance | F_(1,33)_ = 5.60; *p=*0.024; *f^2^*=0.17 | The ε3ε4 carriers had a longer wayfinding distance than ε3ε3 carriers. Cohen’s f2 denoted the R^2^-change from a model with covariates to a model with APOE genotype |
| Laczó et.al., (2021) | Navigation Test Suite | Route-repetition task | F(2,193) = 24.12; *p*<.001, η_p_^2^=0.20 | Repeated-measures ANCOVA with diagnostic group (between-subjects factor) and the session (within-subject factor) were used to assess spatial navigation performance |
|  |  | Route-retracing task | F(2,194) = 14.99; *p*<.001, η_p_^2^=0.13 |  |
|  |  | Directional-Approach task | F(2,193) = 18.21; *p*<.001, η_p_^2^=0.16 |  |
| Castegnaro et.al., (2022) | Object Location Task | Absolute error distance between the real and replaced location of the objects | β_1_ = .64 (CI_95%_ 0.33–1.01); | Linear mixed-effect models were used to assess the group's performance on the task. MCI cohort exhibited larger absolute error distance than the older HC |
|  |  |  | t [1,50] = 3.4; *p* < .001 |  |
| Da Costa et.al., (2022) | SOIVET | Route task - immediate phase | U=164.50; *p* = .009 | The control group significantly outperformed the MCI group in the measures. Groups performances were analyzed using the Wilcoxon Mann-Whitney test |
|  |  | Route task - delayed phase | U=197.00; *p* = .044 |  |
| Laczó et.al., (2022) | Navigation Test Suite | Route-repetition task | F(3,122) = 20.67; *p* <.001, η_p_^2^=0.34 | Mixed-model ANCOVA with diagnostic group (between-subjects factor) and the session or approach direction (within-subject factor) were used to compare the performance |
|  |  | Route-retracing task | F(3,121) = 12.83; *p* <.001, η_p_^2^=0.24 |  |
|  |  | Directional-Approach task | F(3,121) = 14.16; p <.001, η_p_^2^=0.26 |  |
| Park (2022) | SCT-VR | Euclidean distance average | t_(90)_=15.16; *p<*.001 | Independent t-tests were used for assessing differences in the outcome measures. HC group outperformed MCI group in spatial navigation |
| Puthusseryppady et al. (2022) | VST | Egocentric orientation | W = 329, *p* < .001, *d* = 2.47 | AD participants had significantly worse performance in the spatial navigation components compared to HC. T-tests or Wilcoxon Rank Sum tests were used depending on whether the variables had a normal/ non-normal distribution |
|  |  | Allocentric orientation | t = − 3.107, *p* = .004, *d* = 1.04 |  |
|  |  | Allocentric heading direction | W = 334.5, *p* < .001, *d* = 2.57 |  |
| Puthusseryppady et.al., (2022) | Sea Hero Quest | Wayfinding level 6 | W = 59, *p* = .0015, *d* = 1.24 | AD participants showed worse performance than HC on the measures allocentric measures and total time to complete level 6 |
|  |  | Duration to complete level | W = 77, *p* = .011, *d* = 1.13 |  |
| Colmant et al., (2023) | The Apple Game | Drop errors differences | t_197_ = 2.12, p = 0.035 | Performance of participants was analyzed using linear mixed models. Version of the paradigm and participant were allocated as random factors in all models. Gender video game experience, and education were covariates in all models. Performance was quantified based on the drop error. ε4 carriers in the 71–80 age group made higher drop errors than noncarriers of the same age, which provided evidence on the pure path integration deficit on participants with increased AD risk. The response distance depended less on the correct distance for ε4 carriers in the 61–70 age group as compared to noncarriers of the same age. |
|  |  | Response distance differences | t_1123_ = −2.42, p = 0.015 |  |
| Pink et al. (2023) | Virtual reality-based path integration task | Distance and angle estimation | N/R | By employing a linear mixed model, the effects of different factors influencing spatial memory performance. Within individual subjects, experimental manipulation was the predictor. At the subject levels, predictors included age, gender, and genotype. Individuals carrying the APOE4 gene variant demonstrated a higher accuracy in estimating distances |
| Plaza-Rosales et al., (2023) | Virtual Morris Water Navigation (VMWN) | Error rate, latency finding the platform, travel speed, length of the route | p < 0.001 | The error rate was significantly higher in eAD patients when compared to controls (Wilcoxon Rank sum test) both across trials and between the groups along all the trials. The eAD group displayed higher resting times than the control group. Accordingly, navigation speed was consequently reduced in the eAD group. The average latency to find the platform also showed differences. |

**Note:** HC = Healthy Controls; MCI = Mild Cognitive Impairment; AD = Alzheimer’s disease; aMCI = Amnestic Mild Cognitive Impairment; VR = Virtual Reality; VST = Virtual Supermarket Test; yVSA = Y-Maze strategy assessment; VREAD = Virtual Reality for early detection of AD; VReoT = Virtual Reorientation Test; CSDLT = Concurrent Spatial Discrimination Task; SCT-VR = Spatial Cognitive Task Based on Virtual Reality; VRNT= Virtual Neighborhood task; VAP-M = Virtual Museum System; CADL = Complex Activities of Daily Living; VR-MST = VR-Maze Spatial task; VR-RMT= VR-Road Map Task; IIV = Intraindividual variability; eAD = Early Alzheimer’s disease.

**Criterion Validity: Results summary.**

| Reference | VRSG-based instrument | Measure | Standard of reference for the classification | Diagnostic accuracy | | |
| --- | --- | --- | --- | --- | --- | --- |
|  |  |  |  | Se | Sp | AUC-ROC (CI_95%_) |
| Bellassen et.al., (2012) | The Starmaze task | Spatial memory | HC Vs. AD and Frontotemporal dementia | 0.81 | 0.69 | 0.82 (0.70-0.94) |
|  |  | Route tracing |  | 0.81 | 0.91 | 0.87 (0.77-0.97) |
|  |  | Sequential navigation |  | 0.86 | 0.83 | 0.89 (0.80-0.98) |
|  |  | Temporal memory |  | 0.88 | 0.91 | 0.94 (0.87-1.00) |
| Caffò et al., (2012) | VReoT | VReoT score | aMCI Vs. HC | 0.80 | 0.94 | 0.90 (0.84-0.96) |
|  |  | Sum of D and E |  | 0.84 | 0.81 | 0.88 (0.81-0.95) |
|  |  | VReoT score | aMCIsd Vs. HC | 0.79 | 0.94 | 0.88 (0.79-0.97) |
|  |  | Sum of D and E |  | 0.82 | 0.81 | 0.87 (0.78-0.96) |
|  |  | VReoT score | aMCImd Vs. HC | 0.83 | 0.94 | 0.93 (0.86-1.00) |
|  |  | Sum of A, C and E |  | 0.78 | 0.87 | 0.91 (0.83-0.99) |
|  |  | VReoT score | aMCImd Vs. aMCIsd | 0.83 | 0.21 | 0.61 (0.45-0.77) |
|  |  | Sum of B and D |  | 0.78 | 0.43 | 0.70 (0.55-0.85) |
| Allison et.al., (2016) | Cognitive Mapping Task | Wayfinding | CN Biomarker – Vs. CN Biomarker + | 0.92 | 0.57 | 0.77 (0.61-0.92) |
|  |  | Route learning |  | 0.92 | 0.39 | 0.55 (0.39-0.71) |
|  |  | Selective reminding |  | 0.83 | 0.37 | 0.56 (0.37-0.76) |
|  |  | Wayfinding | CN biomarker – Vs. Early-stage Symptomatic AD | 0.92 | 0.71 | 0.89 (0.77-1.00) |
|  |  | Route learning |  | 0.67 | 0.87 | 0.77 (0.61-0.95) |
|  |  | Selective reminding |  | 0.83 | 1.00 | 0.91 (0.77-1.00) |
|  |  | Wayfinding | CN biomarker + Vs. Early Symptomatic AD | 0.67 | 0.92 | 0.79 (0.61-0.96) |
|  |  | Route learning |  | 0.75 | 0.75 | 0.79 (0.61-0.96) |
|  |  | Selective reminding |  | 0.83 | 0.85 | 0.82 (0.62-1.00) |
| Allison et.al., (2019) | Modified cognitive mapping task | Learning phase | Low Αβ_42_ Vs. high Αβ_42_ | 0.93 | 0.60 | 0.72 (0.53-0.91) |
|  |  | Retrieval phase |  | 0.67 | 0.73 | 0.73 (0.55-0.92) |
|  |  | Free recall |  | 0.67 | 0.53 | 0.62 (0.41-0.82) |
|  |  | Landmark identification |  | 0.80 | 0.67 | 0.77 (0.60-0.94) |
|  |  | Scene recognition |  | 0.73 | 0.73 | 0.74 (0.56-0.92) |
| Coughlan et.al., (2019) | Sea Hero Quest | Wayfinding | Carriers Vs. Non-carriers | N/R | N/R | 0.71 (0.55-0.82) |
| Levine et.al., (2020) | Cognitive Mapping Task | Cognitive mapping | Conversion from Global CDR=0 to CDR>0 | 1.00 | 0.71 | 0.86 (0.73-0.99) |
|  | Route Learning Task | Route learning |  | 0.8 | 0.57 | 0.65 (0.44-0.86) |
| Castegnaro et.al., (2022) | Object Location Task | Performance in object replacement subtask | MCI Vs. Older healthy controls | N/R | N/R | 0.89 (0.72-0.95) |
|  |  | Logistic model using OLT-location memory, OLT-immediate free, OLT-object-in-context |  | N/R | N/R | 0.98 (0.94-0.99) |
| Da Costa et.al., (2022) | SOIVET | SOVIET Maze | MCI Vs. HC | 0.74 | 0.62 | 0.73 (0.58-0.84) |
|  |  | SOIVET Route immediate |  | 0.63 | 0.69 | 0.70 (0.55-0.83) |
|  |  | SOIVET Route delayed |  | 0.47 | 0.79 | 0.64 (0.49-0.78) |
| Laczó et.al., (2022) | Navigation Test Suite | Route-repetition | HC vs. non-AD aMCI | 0.67 | 0.65 | 0.72 (0.59-0.85) |
|  |  | Route-retracing |  | 0.73 | 0.60 | 0.68 (0.56-0.83) |
|  |  | Directional approach |  | 0.80 | 0.60 | 0.78 (0.59-0.85) |
|  |  | Route-repetition | HC vs. AD aMCI | 0.87 | 0.79 | 0.89 (0.81-0.98) |
|  |  | Route-retracing |  | 0.80 | 0.73 | 0.86 (0.77-0.95) |
|  |  | Directional approach |  | 0.80 | 0.85 | 0.81 (0.69-0.92) |
|  |  | Route-repetition | HC vs. mild AD dementia | 0.87 | 0.82 | 0.92 (0.85-0.99) |
|  |  | Route-retracing |  | 0.80 | 0.71 | 0.89 (0.81-0.97) |
|  |  | Directional approach |  | 0.80 | 0.93 | 0.88 (0.79-0.97) |
|  |  | Route-repetition | Non-AD aMCI vs. AD aMCI | 0.68 | 0.70 | 0.78 (0.66-0.89) |
|  |  | Route-retracing |  | 0.67 | 0.52 | 0.64 (0.51-0.78) |
|  |  | Directional approach |  | 0.57 | 0.75 | 0.62 (0.47-0.76) |
|  |  | Route-repetition | Non-AD aMCI vs. mild AD | 0.68 | 0.71 | 0.80 (0.69-0.91) |
|  |  | Route-retracing |  | 0.67 | 0.57 | 0.65 (0.51-0.80) |
|  |  | Directional approach |  | N/R | N/R | 0.71 (0.58-0.84) |
|  |  | Route-repetition | AD aMCI vs. mild AD dementia | N/R | N/R | 0.63 (0.49-0.77) |
|  |  | Route-retracing |  | N/R | N/R | 0.50 (0.35-0.65) |
|  |  | Directional approach |  | N/R | N/R | 0.60 (0.46-0.74) |
| Park et.al., (2022) | SCT-VR | Distance error | MCI Vs. Healthy Aging | 0.94 | 0.96 | 0.91 (0.84-0.98) |

**Note:** AUC-ROC = Area Under the ROC Curve; Se = Sensitivity; Sp = Specificity; MCI = Mild cognitive impairment; AD = Alzheimer’s disease; aMCI = Amnesic mild cognitive impairment; HC = Healthy Controls; CN = Cognitively normal.

**Construct validity evidence: Structural validity.**

**Confirmatory factor analysis.**

Confirmatory factor analysis was done to test whether the spatial navigation-related tasks converged to form one latent factor.

| Reference | VRSG-based instrument | Factor Analysis | | | |
| --- | --- | --- | --- | --- | --- |
|  |  | **Chi-squared** | **CFI** | **RMSEA (CI_90%_)** | **SRMR** |
| Allison et.al., (2019) | Modified cognitive mapping task | Χ2(14) = 18.17; p = .199 | 0.904 | 0.06 (0.00-0.12) | 0.064 |

**Exploratory factor analysis.**

Factor analysis using all neuropsychological and experimental tests was performed. Four factors with eigenvalues >1, which explained 72% of the total variance, were found. The first factor (i.e., Memory) explained 47% of the variance and included verbal memory subtests, temporal memory scores, and spatial memory tests. This factor constitutes a simple index of Memory. Other factors corresponding to general cognitive and visuoconstructive functions, encoding of the environment, and executive function, explained 10% variance each. Factor analysis after varimax rotation on all variables is shown below:

| Reference | VRSG-based instrument |  | First factor | Second factor | Third factor | Fourth factor |
| --- | --- | --- | --- | --- | --- | --- |
| Bellassen et.al., (2012) | The Starmaze task | *First factor: Memory* | | | | |
|  |  | FCSRT-FR | 0.84 | 0.13 | -0.01 | 0.33 |
|  |  | FCSRT-TR | 0.93 | 0.11 | -0.06 | 0.21 |
|  |  | FCSRT-CS | 0.93 | 0.10 | -0.06 | 0.22 |
|  |  | Temporal memory | 0.73 | 0.40 | 0.18 | 0.09 |
|  |  | Spatial memory | 0.54 | 0.40 | -0.32 | 0.05 |
|  |  | *Second factor: General cognitive and visuoconstructive functions* | | | | |
|  |  | % Successful trials | 0.15 | 0.81 | -0.07 | 0.10 |
|  |  | RCFT copy | 0.16 | 0.61 | 0.00 | 0.59 |
|  |  | MMSE-O | 0.55 | 0.57 | 0.03 | 0.19 |
|  |  | MMSE-R | 0.38 | 0.59 | 0.04 | 0.26 |
|  |  | MMSE | 0.54 | 0.50 | -0.07 | 0.50 |
|  |  | % Direct trials | 0.22 | 0.54 | 0.44 | -0.14 |
|  |  | Exploration | 0.15 | -0.46 | 0.23 | -0.42 |
|  |  | *Third factor: Encoding the environment* |  |  |  |  |
|  |  | “What” | 0.09 | 0.04 | -0.90 | -0.10 |
|  |  | Fourth factor: Executive function |  |  |  |  |
|  |  | FAB | 0.35 | 0.04 | 0.08 | 0.82 |
|  |  | CBT-F | 0.46 | 0.25 | 0.13 | 0.61 |
|  |  | CBT-B | 0.59 | 0.09 | 0.03 | 0.70 |
|  |  | % of variance | 47% | 10% | 8% | 7% |

**Note:** MMSE = Minimental State Examination; RCFT = Rey complex figure test; FCSRT = free and cued selective reminding test; FAB = Frontal Assessment Battery; CBT-B = Corsi block-tapping task – Backward; CBT-F = Corsi block-tapping task – Forward.

**References**

Allison, S. L., Fagan, A. M., Morris, J. C. & Head, D. (2016). Spatial Navigation in Preclinical Alzheimer’s Disease Samantha. *Journal of Alzheimer’s Disease*, *52*(1), 77–90. <https://doi.org/10.3233/JAD-150855>.

Allison, S. L., Rodebaugh, T. L., Johnston, C., Fagan, A. M., Morris, J. C. & Head, D. (2019). Developing a Spatial Navigation Screening Tool Sensitive to the Preclinical Alzheimer Disease Continuum. *Archives of Clinical Neuropsychology*, *34*(7), 1138–1155. <https://doi.org/10.1093/arclin/acz019>

Bayahya, A. Y., Alhalabi, W. & Alamri, S. H. (2021). Smart health system to detect dementia disorders using virtual reality. *Healthcare (Switzerland)*, *9*(7) 810. <https://doi.org/10.3390/healthcare9070810>

Bellassen, V., Iglói, K., de Souza, L. C., Dubois, B. & Rondi-Reig, L. (2012). Temporal order memory assessed during spatiotemporal navigation as a behavioral cognitive marker for differential Alzheimer’S disease diagnosis. *Journal of Neuroscience*, *32*(6), 1942–1952. <https://doi.org/10.1523/JNEUROSCI.4556-11.2012>

Bierbrauer, A., Kunz, L., Gomes, C. A., Luhmann, M., Deuker, L., Getzmann, S., Wascher, E., Gajewski, P. D., Hengstler, J. G., Fernandez-Alvarez, M., Atienza, M., Cammisuli, D. M., Bonatti, F., Pruneti, C., Percesepe, A., Bellaali, Y., Hanseeuw, B., Strange, B. A., Cantero, J. L. & Axmacher, N. (2020). Unmasking selective path integration deficits in Alzheimer’s disease risk carriers. *Science Advances, 6*(35). <https://doi.org/10.1126/sciadv.aba1394>

Caffò, A. O., De Caro, M. F., Picucci, L., Notarnicola, A., Settanni, A., Livrea, P., Lancioni, G. E. & Bosco, A. (2012). Reorientation deficits are associated with amnestic mild cognitive impairment. *American Journal of Alzheimer’s Disease and Other Dementias*, *27*(5), 321–330.

Caffò, A. O., Lopez, A., Spano, G., Serino, S., Cipresso, P., Stasolla, F., Savino, M., Lancioni, G. E., Riva, G. & Bosco, A. (2018). Spatial reorientation decline in aging: the combination of geometry and landmarks. *Aging and Mental Health, 22*(10), 1372–1383. <https://doi.org/10.1080/13607863.2017.1354973>

Castegnaro, A., Howett, D., Li, A., Harding, E., Chan, D., Burgess, N. & King, J. (2022). Assessing mild cognitive impairment using object-location memory in immersive virtual environments. *Hippocampus*, *32*(9), 660–678. <https://doi.org/10.1002/hipo.23458>

Colmant, L., Bierbrauer, A., Bellaali, Y., Kunz, L., Van Dongen, J., Sleegers, K., Axmacher, N., Lefèvre, P. & Hanseeuw, B. (2023). Dissociating effects of aging and genetic risk of sporadic Alzheimer’s disease on path integration. *Neurobiology of Aging, (131)*, 170–181. <https://doi.org/10.1016/j.neurobiolaging.2023.07.025>

Coughlan, G., Coutrot, A., Khondoker, M., Minihane, A. M., Spiers, H. & Hornberger, M. (2019). Toward personalized cognitive diagnostics of at-genetic-risk Alzheimer’s disease. *Proceedings of the National Academy of Sciences of the United States of America*, *116*(19), 9285–9292. <https://doi.org/10.1073/pnas.1901600116>

Da Costa, R. Q. M., Pompeu, J. E., Moretto, E., Silva, J. M., Dos Santos, M. D., Nitrini, R. & Brucki, S. M. D. (2022). Two Immersive Virtual Reality Tasks for the Assessment of Spatial Orientation in Older Adults with and Without Cognitive Impairment: Concurrent Validity, Group Comparison, and Accuracy Results. *Journal of the International Neuropsychological Society*, *28*(5), 460–472. <https://doi.org/10.1017/S1355617721000655>

Davis, R. & Sikorskii, A. (2020). Eye Tracking Analysis of Visual Cues during Wayfinding in Early Stage Alzheimer’s Disease. *Dementia and Geriatric Cognitive Disorders*, *49*(1), 91–97. <https://doi.org/10.1159/000506859>

Gellersen, H. M., Coughlan, G., Hornberger, M. & Simons, J. S. (2021). Memory precision of object-location binding is unimpaired in APOE ϵ4-carriers with spatial navigation deficits. *Brain Communications*, *3*(2). <https://doi.org/10.1093/braincomms/fcab087>

Konishi, K., Joober, R., Poirier, J., MacDonald, K., Chakravarty, M., Patel, R., Breitner, J. & Bohbot, V. D. (2018). Healthy versus entorhinal cortical atrophy identification in asymptomatic APOE4 carriers at risk for Alzheimer’s disease. *Journal of Alzheimer’s Disease*, *61*(4), 1493–1507. <https://doi.org/10.3233/JAD-170540>

Laczó, M., Martinkovic, L., Lerch, O., Wiener, J. M., Kalinova, J., Matuskova, V., Nedelska, Z., Vyhnalek, M., Hort, J. & Laczó, J. (2022). Different Profiles of Spatial Navigation Deficits In Alzheimer’s Disease Biomarker-Positive Versus Biomarker-Negative Older Adults With Amnestic Mild Cognitive Impairment. *Frontiers in Aging Neuroscience*, *14*. <https://doi.org/10.3389/fnagi.2022.886778>

Laczó, M., Wiener, J. M., Kalinova, J., Matuskova, V., Vyhnalek, M., Hort, J. & Laczó, J. (2021). *Spatial Navigation and Visuospatial Strategies in Typical and Atypical Aging*. <https://doi.org/10.3390/brainsci>

Lee, J., Kho, S., Yoo, H. Bin, Park, S. & Choi, J. (2014). Spatial memory impairments in amnestic mild cognitive impairment in a virtual radial arm maze. *Neuropsychiatric Disease and Treatment*, *10*, 653–660. <https://doi.org/10.2147/NDT.S58185>

Lesk, V. E., Wan Shamsuddin, S. N., Walters, E. R. & Ugail, H. (2014). Using a virtual environment to assess cognition in the elderly. *Virtual Reality*, *18*(4), 271–279. <https://doi.org/10.1007/s10055-014-0252-2>

Levine, T. F., Allison, S. L., Stojanovic, M., Fagan, A. M., Morris, J. C. & Head, D. (2020). Spatial navigation ability predicts progression of dementia symptomatology. *Alzheimer’s and Dementia*, *16*(3), 491–500. <https://doi.org/10.1002/alz.12031>

Migo, E. M., O’Daly, O., Mitterschiffthaler, M., Antonova, E., Dawson, G. R., Dourish, C. T., Craig, K. J., Simmons, A., Wilcock, G. K., McCulloch, E., Jackson, S. H. D., Kopelman, M. D., Williams, S. C. R. & Morris, R. G. (2016). Investigating virtual reality navigation in amnestic mild cognitive impairment using fMRI. *Neuropsychology, Development, and Cognition. Section B, Aging, Neuropsychology and Cognition*, *23*(2), 196–217. <https://doi.org/10.1080/13825585.2015.1073218>

Mohammadi, A., Kargar, M. & Hesami, E. (2018). Using virtual reality to distinguish subjects with multiple- but not single-domain amnestic mild cognitive impairment from normal elderly subjects. *Psychogeriatrics*, *18*(2), 132–142. <https://doi.org/10.1111/psyg.12301>

Morganti, F., Stefanini, S. & Riva, G. (2013). From allo- to egocentric spatial ability in early Alzheimer’s disease: A study with virtual reality spatial tasks. *Cognitive Neuroscience*, *4*(3–4), 171–180. <https://doi.org/10.1080/17588928.2013.854762>

Parizkova, M., Lerch, O., Moffat, S. D., Andel, R., Mazancova, A. F., Nedelska, Z., Vyhnalek, M., Hort, J. & Laczó, J. (2018). The effect of Alzheimer’s disease on spatial navigation strategies. *Neurobiology of Aging*, *64*, 107–115. <https://doi.org/10.1016/j.neurobiolaging.2017.12.019>

Park, J. H. (2022). Can the Virtual Reality-Based Spatial Memory Test Better Discriminate Mild Cognitive Impairment than Neuropsychological Assessment? *International Journal of Environmental Research and Public Health*, *19*(16). <https://doi.org/10.3390/ijerph19169950>

Pink, D., Ilkel, E., Chandreswaran, V., Moser, D., Getzmann, S., Patrick, G., Axmacher, N. & Zhang, H. (2023). Modeling the impact of genotype, age, sex, and continuous navigation on pathway integration performance. *BioRxiv.* <https://doi.org/10.1101/2023.09.11.556925>

Plaza-Rosales, I., Brunetti, E., Montefusco-Siegmund, R., Madariaga, S., Hafelin, R., Ponce, D. P., Behrens, M. I., Maldonado, P. E., & Paula-Lima, A. (2023). Visual-spatial processing impairment in the occipital-frontal connectivity network at early stages of Alzheimer’s disease. *Frontiers in Aging Neuroscience, 15*. <https://doi.org/10.3389/fnagi.2023.1097577>

Puthusseryppady, V., Morrissey, S., Spiers, H., Patel, M. & Hornberger, M. (2022). Predicting real world spatial disorientation in Alzheimer’s disease patients using virtual reality navigation tests. *Scientific Reports*, *12*(1). <https://doi.org/10.1038/s41598-022-17634-w>

Ritchie, K., Carrière, I., Howett, D., Su, L., Hornberger, M., O’Brien, J. T., Ritchie, C. W. & Chan, D. (2018). Allocentric and egocentric spatial processing in middle-aged adults at high risk of late-onset Alzheimer’s disease: The PREVENT dementia study. *Journal of Alzheimer’s Disease*, *65*(3), 885–896. <https://doi.org/10.3233/JAD-180432>

Serino, S., Morganti, F., Di Stefano, F. & Riva, G. (2015). Detecting early egocentric and allocentric impairments deficits in Alzheimer’s disease: an experimental study with virtual reality. *Frontiers in Aging Neuroscience*, *7*, 1–10. <https://doi.org/10.3389/fnagi.2015.00088>

Serino, S., Morganti, F., Colombo, D. & Riva, G. (2018). The Contribution of Allocentric Impairments to the Cognitive Decline in Alzheimer’s Disease. *Lecture Notes of the Institute for Computer Sciences, Social-Informatics and Telecommunications Engineering, LNICST*, *253*, 84–91. <https://doi.org/10.1007/978-3-030-01093-5_11>

Silva, J. M. da, Santos, M. D. dos, Costa, R. Q. M. da, Moretto, E. G., Viveiro, L. A. P. de, Lopes, R. de D., Brucki, S. M. D. & Pompeu, J. E. (2023). Applicability of an immersive virtual reality system to assess egocentric orientation of older adults. *Arquivos de Neuro-Psiquiatria, 81*(01), 019–026. <https://doi.org/10.1055/s-0042-1759762>

Tarnanas, I., Laskaris, N. & Tsolaki, M. (2012). On the comparison of VR-responses, as performance measures in prospective memory, with auditory P300 responses in MCI detection. *Studies in health technology and informatics, 181,* 156–161.

Tarnanas, I., Papagiannopoulos, S., Kazis, D., Wiederhold, M., Widerhold, B., Vuillermot, S. & Tsolaki, M. (2015). Reliability of a novel serious game using dual-task gait profiles to early characterize aMCI. *Frontiers in Aging Neuroscience*, *7*. <https://doi.org/10.3389/fnagi.2015.00050>

Zen, D., Byagowi, A., Tere Garcia Campuzano, M., kelly, D., Lithgow, B. & Moussavi, Z. (2013). *The Perceived Orientation in People with and without Alzheimer´s*. <https://doi.org/10.1109/ner.2013.6695971>
