# Supplementary material for: Virtual Reality and Serious Videogame-Based Instruments for Assessing Spatial Navigation in Alzheimer’s Disease: A Systematic Review of Psychometric Properties
Source: Neuropsychol Rev. 2024 Feb 26;35(1):77–101. doi: 10.1007/s11065-024-09633-7 (PMC11965194; doi:10.1007/s11065-024-09633-7)
Supplement: Supplementary file 3 — Supplementary file3 (DOCX 16 KB) [file 11065_2024_9633_MOESM3_ESM.docx]

**Supplementary Material.**

**Recommendations for the development and improvement of Virtual Reality and Serious Games-based instruments.**

The following recommendations stem from a comprehensive analysis of the existing evidence concerning the psychometric properties of Virtual Reality Spatial Navigation (VRSG) instruments used for assessing spatial navigation in individuals with Alzheimer's Disease in different stages. The primary aim of these recommendations is to offer guidance for the refinement and enhancement of current instruments within this field and the development of new instruments with the highest standards of psychometric quality.

| Recommendation | Description |
| --- | --- |
| *Consider using the cognitive map hypothesis as your theoretical framework* | The cognitive map hypothesis, widely recognized as the most supported theoretical framework in the literature on spatial navigation, provides the essential elements for the development of tasks that hold significant value in the context of preclinical research on AD. Particularly the distinction between allocentric and egocentric strategies has showed been helpful in the development of discriminant measures for prodromic AD. |
| *Declare the purpose of the correlation analysis.* | To ensure accurate interpretation of results, it is crucial to explicitly state the purpose and anticipated outcomes of correlation analyses prior to conducting them. |
| *Use biomarkers as standard of reference for preclinical AD.* | According to the NIAA research framework, although different sources of evidence are accepted to determine the AD stage, only biomarkers (i.e., Αβ and tau markers) are assumed to determine the presence of AD since the preclinical stage. Follow the recommendations of this framework for classifying the participants in your research. |
| *Test the usability of the instrument* | Incorporating pilot studies can be beneficial in assessing the usability of your instrument. Consider implementing usability questionnaires to gather valuable feedback during the design process. |
| *Publish the results of pilot studies* | Whenever feasible, it is advisable to publish the results of usability testing and pilot studies. Sharing this information can contribute to the advancement of new paradigms and accelerate research in the field. |
